# Characterizing the spatiotemporal features of functional connectivity across the white matter and gray matter during the naturalistic condition

**DOI:** 10.3389/fnins.2023.1248610

**Published:** 2023-11-09

**Authors:** Peng Hu, Pan Wang, Rong Zhao, Hang Yang, Bharat B. Biswal

**Affiliations:** ^1^MOE Key Laboratory for Neuroinformation, The Clinical Hospital of Chengdu Brain Science Institute, Center for Information in Medicine, School of Life Science and Technology, University of Electronic Science and Technology of China, Chengdu, China; ^2^Chinese Institute for Brain Research, Beijing, China; ^3^Department of Biomedical Engineering, New Jersey Institute of Technology, Newark, NJ, United States

**Keywords:** functional connectivity, inter-subject functional connectivity, intra-class correlation coefficient, naturalistic condition, white matter

## Abstract

**Introduction:**

The naturalistic stimuli due to its ease of operability has attracted many researchers in recent years. However, the influence of the naturalistic stimuli for whole-brain functions compared with the resting state is still unclear.

**Methods:**

In this study, we clustered gray matter (GM) and white matter (WM) masks both at the ROI- and network-levels. Functional connectivity (FC) and inter-subject functional connectivity (ISFC) were calculated in GM, WM, and between GM and WM under the movie-watching and the resting-state conditions. Furthermore, intra-class correlation coefficients (ICC) of FC and ISFC were estimated on different runs of fMRI data to denote the reliability of them during the two conditions. In addition, static and dynamic connectivity indices were calculated with Pearson correlation coefficient to demonstrate the associations between the movie-watching and the resting-state.

**Results:**

As the results, we found that the movie-watching significantly affected FC in whole-brain compared with the resting-state, but ISFC did not show significant connectivity induced by the naturalistic condition. ICC of FC and ISFC was generally higher during movie-watching compared with the resting-state, demonstrating that naturalistic stimuli could promote the reliability of connectivity. The associations between static and dynamic ISFC were weakly negative correlations in the naturalistic stimuli while there is no correlation between them under resting-state condition.

**Discussion:**

Our findings confirmed that compared to resting-state condition, the connectivity indices under the naturalistic stimuli were more reliable and stable to investigate the normal functional activities of the human brain, and might promote the applications of FC in the cerebral dysfunction in various mental disorders.

## 1. Introduction

Functional connectivity (FC) was proposed as a measure to characterize the temporal synchronization between different brain regions (Aertsen et al., 1989; Friston et al., 1993). Biswal and colleagues found that primary motor network existed during the resting-state by performing FC, suggesting that the human brain has also functional pattern even though subjects did not perform a specific task (Biswal et al., 1995). Additional intrinsic function networks have also been extracted from resting-state fMRI (rsfMRI) adopting the FC measure, such as the auditory network (Cordes et al., 2000), visual network (Lowe et al., 1998), and default mode network (Greicius et al., 2003). Furthermore, FC under rsfMRI has been associated with demographic and cognitive measures, such as age (Dosenbach et al., 2010), gender (Zhang et al., 2016, 2018b), and fluid intelligence (Finn et al., 2015). FC during rsfMRI has also provided an impetus to the field of brain disorders, such as autism (Plitta et al., 2015), schizophrenia (Yang et al., 2018), Alzheimer (Dennis and Thompson, 2014), and depression (Villa et al., 2020). These studies have established that FC measures have many important applications in characterizing the human brain’s functional activities.

Since static FC (sFC) did not consider the dynamic of signal changes in time, researchers further investigate the dynamic fluctuation of FC (Chang et al., 2009). Dynamic FC (dFC) also have important applications in recognizing relationship between the behavioral/clinical measures and brain functional activities such as the attention scores (Fong et al., 2019), brain maturity (Qin et al., 2015) and cognitive vulnerabilities unmasked by a stressor like sleep restriction (Patanaik et al., 2018). Moreover, combining dFC and sFC features numerically improves predictions over either model alone (Fong et al., 2019). dFC hold promise to provide fundamental information for the neurodegenerative diseases, including Alzheimer’s disease (Jones et al., 2012; Córdova-Palomera et al., 2017; Jie et al., 2018), Parkinson’s disease (Madhyastha et al., 2015; Kim et al., 2017; Liu et al., 2018), and dementia with Lewy bodies (Lowther et al., 2014; Peraza et al., 2014). The aforementioned studies suggested that the dFC is as important as the sFC for understanding the functional activity of the human brain and psychiatric disorders.

Accumulating naturalistic stimuli studies including watching the movie and listening to the story have attracted the interest of many researchers as a compromise between rsfMRI and task-evoked fMRI (tfMRI). Compared with rsfMRI, naturalistic condition improves the similarity between subjects as they are experiencing the same stimuli (Hasson et al., 2004; Nastase et al., 2019). With continuous presentation of sound and visual information, naturalistic scanning is closer to the real life environment (Chen G. et al., 2020). Furthermore, previous studies demonstrated that the naturalistic paradigm was better than the resting-state for controlling the head motion, especially for children (Vanderwal et al., 2015, 2019; Greene et al., 2018). Movie-watching paradigm also has been shown to improve participant arousal levels (Vanderwal et al., 2017). Compared with tfMRI, naturalistic condition is easier to perform particularly for children, elders, and clinical populations (Vanderwal et al., 2015; Huijbers et al., 2017).

As both movie-watching and resting-state had no specific task when subjects were scanned, the naturalistic paradigm is more similar to rsfMRI than tfMRI, resulting in the universality of FC patterns that are especially associated with the resting-state. Finn and Bandettini demonstrated that compared to the resting-state condition, FC during the naturalistic viewing (i.e., movie watching) gave more accurate prediction of trait-like phenotypes in the domains of both cognition and emotion (Finn and Bandettini, 2021). A widespread FC pattern was identified that it could predict whether individuals are watching a movie or resting (Sanchez-Alonso et al., 2021). To uncover differences between two states, Lynch and colleagues calculated the FC differences between them, and suggested that the naturalistic condition showed weaker FC than the resting-state after correction (Lynch et al., 2018). However, only 10 subjects were used in this study and the length of the movie clip was less than 6 min, suggesting that their findings need to be evaluated in a large group of subjects. In another study, the FC reliability of brain networks was significantly improved during natural viewing conditions over resting-state conditions, with an average increase of almost 50% across various connectivity measures (Wang et al., 2017).

The dFC measure has recently been used in the naturalistic stimuli as a method of detecting the neuro-information (Sastry et al., 2022). For clinical populations involving body dysmorphic disorder (BDD), Wong and colleagues uncovered that the naturalistic viewing could affect dynamic connectivity when symptoms of BDD were triggered (Wong et al., 2021). Previous studies have shown that although dFC values exhibited weaker reliability than sFC during the resting-state and in the naturalistic viewing condition, the reliability of dFC could be significantly improved in the naturalistic viewing (Zhang et al., 2018a, 2021; Wang et al., 2021).

A basic question in neuroscience is how synchronous different subjects’ brains are under the real-world. To answer this question, the naturalistic condition such as movie have been used to simulate the real-world stimuli. Inter-subject correlation (ISC) and inter-subject functional connectivity (ISFC) have been performed to evaluate the synchronization between subjects. Hasson and colleagues calculated ISC during natural vision and found that similarities for visual, auditory, and their association cortices were significantly improved (Hasson et al., 2004). Furthermore, dynamic ISC was proposed to explore the consistent dynamic connectivity between different subjects in widespread brain regions (Di and Biswal, 2020). As an extension of ISC, Simony and colleagues introduced ISFC that was calculated between the time series in one ROI of one subject and the average time series in all ROIs of all subjects. ISFC could be used to evaluate stimuli-evoked correlations, because non-neuronal artificial signals are not correlated between different subjects (Simony et al., 2016). Moreover, ISFC could be calculated with another way. Specifically, the correlation was evaluated between the time series of one ROI of one subject and all ROIs’ time series across other subjects. The approach of correlating each subject with every other subject (rather than correlating each subject’s data with all other subjects’ data average) preserves the variability from individual subjects (Cantlon and Li, 2013). The authors found that primary sensory cortices appeared to have strong ISFC consistence (Ren et al., 2017). Furthermore, ISFC was proved as an effective measure to evaluate specific brain disorder, such as attention deficit hyperactivity disorder (ADHD; Tang et al., 2019), and had benefit to discover the influence of negative family environments for children’s psychological wellbeing (Su et al., 2022). The joint model (ISFC + FC) yield the highest predictive accuracy and significantly predicted individuals’ social cognitive abilities. The model also proved the hypothesize that ISFC and FC provide distinct and complementary information about individual differences in social cognition (Xie and Redcay, 2022). ISFC has ability to explore the importance of feed-back connections in action prediction (Cerliani et al., 2022). Dynamic ISFC (dISFC) was able to isolate effects of the naturalistic condition and to capture the temporal information of brain activity (Bolton et al., 2019). Furthermore, dISFC could be used to detect the functional brain configurations, which autism spectrum disorder and typical development subjects continuously adjusted based on movie cues under the naturalistic stimulation (Bolton et al., 2020). The effect of naturalistic stimuli for FC and ISFC compared with rsfMRI has not been systematically addressed.

White matter (WM) is a dense structure that connects to different gray matter (GM) areas and occupies almost half of human brain (Teo et al., 1997; Zhang and Sejnowski, 2000; Arai and Lo, 2009; Harris and Attwell, 2012). Although the WM plays an important role in the human brain, the functional properties of WM were rarely analyzed and frequently ignored in fMRI. Recent studies have suggested that WM shows blood oxygen level-dependent (BOLD) signal fluctuations similar to those of GM (Peer et al., 2017; Jiang et al., 2019; Ji F. et al., 2019; Ji G. J. et al., 2019). Peer and colleagues found that the signals of WM could be organized with FC to generate symmetrical WM functional networks and the replication was proved in an independent group (Peer et al., 2017; Wang et al., 2021). Furthermore, by evaluating the reliability of sFC and dFC in GM and WM, Wang and colleagues found that the reliability of sFC was higher than that of dFC, applying to both WM and GM (Wang et al., 2021). Moreover, WM functional signals could be used to explain neurological diseases, such as schizophrenia (Jiang et al., 2019; Fan et al., 2020), Parkinson’s disease (Ji G. J. et al., 2019), and Alzheimer’s disease (Ji F. et al., 2019). However, the influence of naturalistic condition for WM functional signals remains unclear.

In this study, we aim to investigate the influence of the naturalistic paradigm on the whole-brain FC compared with the resting state. We have mainly focused on the following three topics: (1) Compared with the resting-state, the differences of naturalistic paradigm for different connectivity in whole-brain (sFC, dFC, sISFC and dISFC in GM, WM, and between GM-WM); (2) Evaluating the reliability of different connectivity in the naturalistic and the resting-state conditions; (3) Associations between the naturalistic stimuli and the resting-state conditions, and associations between them for different connectivity measures. Specifically, we clustered GM templates with 200 ROIs and 8 functional networks and adopted similar processing steps to obtain WM templates with 200 ROIs and 9 functional networks. sFC, dFC, sISFC, and dISFC were calculated for both movie-watching and resting-state at ROI- and network-levels. To compare sFC and dFC between the naturalistic and resting-state conditions, we calculated paired-T test to show significant changes between two states. The subject-wise bootstrapping (SWB) was performed to show the significant sISFC and dISFC induced by naturalistic viewing. ICC was performed to demonstrate the reliability of FC and ISFC. Heat maps were calculated to denote associations between two conditions and between static and dynamic properties.

## 2. Materials and methods

### 2.1. Human connectome project data

This study used data from the WU-Minn 7-Tesla Human Connectome Project (HCP). This is a widely used open-access dataset.^1^ Using the 7-Tesla scanner a total of 184 subjects were scanned. Eight subjects had incomplete data and were therefore excluded from this study. We also removed four subjects with missing rest or movie images. Therefore, a total of 172 subjects could be used in this study. Briefly, in this study, four sessions were scanned across two or three different days. A number of imaging sequences including movie-watching and resting-state data was performed. Specifically, session1 included MOVIE1, MOVIE2, and REST1; session2 included REST2; session3 included REST3; and session4 included MOVIE3, MOVIE4, and REST4. The REST and MOVIE runs were collected using the same gradient-echo-planar imaging (EPI) sequence with the following parameters: repetition time (TR) = 1,000 ms, echo time (TE) = 22.2 ms, flip angle = 45 deg., field of view (FOV) = 208 × 208 mm, matrix = 130 × 130, spatial resolution = 1.6 mm^3^, number of slices = 85, multiband factor = 5, image acceleration factor = 2, partial fourier sampling = 7/8, echo spacing = 0.64 ms, bandwidth = 1,924 Hz/Px. The parameters of T1 series were as following: TR = 2,400 ms, TE = 2.14 ms, TI = 1,000 ms, flip angle = 8 deg., FOV = 224 × 224 mm, voxel size = 0.7 mm isotropic, BW = 210 Hz/Px, iPAT = 2, acquisition time = 7 min and 40 s. The direction of phase encoding alternated between posterior-to-anterior (REST1, REST2, MOVIE2, MOVIE3) and anterior-to-posterior (REST3, REST4, MOVIE1, MOVIE4). Frames (TRs, second) per run for REST = 900; frames of MOVIE1 = 921; frames of MOVIE2 = 918; frames of MOVIE3 = 915; frames of MOVIE4 = 901.

Detailed information about the Movie paradigm has been described elsewhere and therefore only briefly described here (Finn and Bandettini, 2021). The MOVIE runs contained several rest and movie clips. There was a rest clip before and after movie clips. The detailed MOVIE clips’ time points were described in Table 1.

**Table 1:** The information about movie runs.

Run	Clip	Start TP	End TP	Duration	Run	Clip	Start TP	End TP	Duration
MOVIE1	1	21	264	244	MOVIE3	1	21	201	181
	2	285	506	222		2	222	406	185
	3	527	714	188		3	427	630	204
	4	735	798	64		4	651	792	142
	5	819	901	83		5	813	895	83
MOVIE2	1	21	247	227	MOVIE4	1	21	253	233
	2	268	526	259		2	274	503	230
	3	547	795	249		3	524	778	255
	4	816	898	83		4	799	881	83

Here are the start time point, end time point, and duration of movie clips for all four movie runs. TP, time point.

### 2.2. The preprocessing steps of fMRI dataset

The preprocessing steps of fMRI data were performed using custom built MATLAB scripts and the Data Processing Assistant for Resting-State fMRI (DPARSF; Chao-Gan and Yu-Feng, 2010)^2^ and Statistical Parametric Mapping (SPM12).^3^ REST and MOVIE data sets were analyzed using the same preprocessing steps. The first 20 time points were discarded as the first clip of MOVE data was a 20s rest clip, and to minimize the T1 relaxation. Head motion was corrected using rigid body translation and rotation. T1-weighted anatomic images were co-registrated with functional images, then segmented to GM, WM, and CSF by using the DARTEL algorithm. Linear drift was detrended. CSF signal as covariates were regressed from functional images. Head motion was also regressed based on Friston 24-parameter model (6 head motion parameters, 6 head motion parameters one time point before, and the 12 corresponding squared items; Friston et al., 1996). To further reduce the influence of head motion, we performed scrubbing regressors by motion “spikes,” which was defined by the frame-wise displacement (FD) > 1 mm and used by previous WM BOLD signals studies demonstrating highly reliable FC both in GM and WM (Power et al., 2012; Peer et al., 2017; Jiang et al., 2019; Wang et al., 2020, 2021). Previous studies demonstrated that the head motion scrubbing did not affect correlation coefficients and could effectively reduce the influence of head motion (Power et al., 2012; Satterthwaite et al., 2013). A temporal filter with range from 0.01 to 0.1 Hz was performed to reduce the non-neuronal contribution to blood oxygen level dependent fluctuations. The functional images were smoothed separately within GM and WM using MATLAB scripts to avoid the confusion between GM and WM signals. Normalization was performed to transform the smoothed functional images to standard Montreal Neurological Institute (MNI) space with 3 × 3 × 3 mm^3^.

After preprocessing the data sets using the steps described above, the resting state clips (4 or 5) were removed from the MOVIE runs. The remaining time points of MOVIE runs were as follows: MOVIE1 = 801 (244 + 222 + 188 + 64 + 83, second); MOVIE2 = 818 (227 + 259 + 249 + 83, second); MOVIE3 = 795 (181 + 185 + 204 + 142 + 83, second); and MOVIE4 = 801 (233 + 230 + 255 + 83, second; Table 1). MOVIE1 and MOVIE2 were concatenated as MOVIE Day1 (time points = 801 + 818 = 1,619, second); MOVIE3 and MOVIE4 were concatenated as MOVIE Day2 (time points = 795 + 801 = 1,596, second); REST1 and REST2 were concatenated as REST Day1 (time points = 880 + 880 = 1,760, second); REST3 and REST4 were concatenated as REST Day2 (time points = 880 + 880 = 1,760, second; Table 2). The advantage of concatenation was to increase signal length to improve signal-noise ratio. Furthermore, except the last movie clip, all other movie clips are different, so it is hard to average MOVIE runs. Though some subjects were not scanned in 2 days but in 3 days, we still separate them in Day1 and Day2 as shown above.

**Table 2:** The description of concatenate MOVIE and REST runs.

Series	Concatenate runs	Time points
MOVIE Day1	MOVIE1 + MOVIE2	801 + 818 = 1,619
MOVIE Day2	MOVIE3 + MOVIE4	795 + 801 = 1,596
REST Day1	REST1 + REST2	880 + 880 = 1,760
REST Day2	REST3 + REST4	880 + 880 = 1,760

MOVIE1 and MOVIE2 were concatenated as MOVIE Day1; MOVIE3 and MOVIE4 were concatenated as MOVIE Day2; REST1 and REST2 were concatenated as REST Day1; REST3 and REST4 were concatenated as REST Day2.

### 2.3. Creation of group-level masks and functional networks

**Figure 1:**
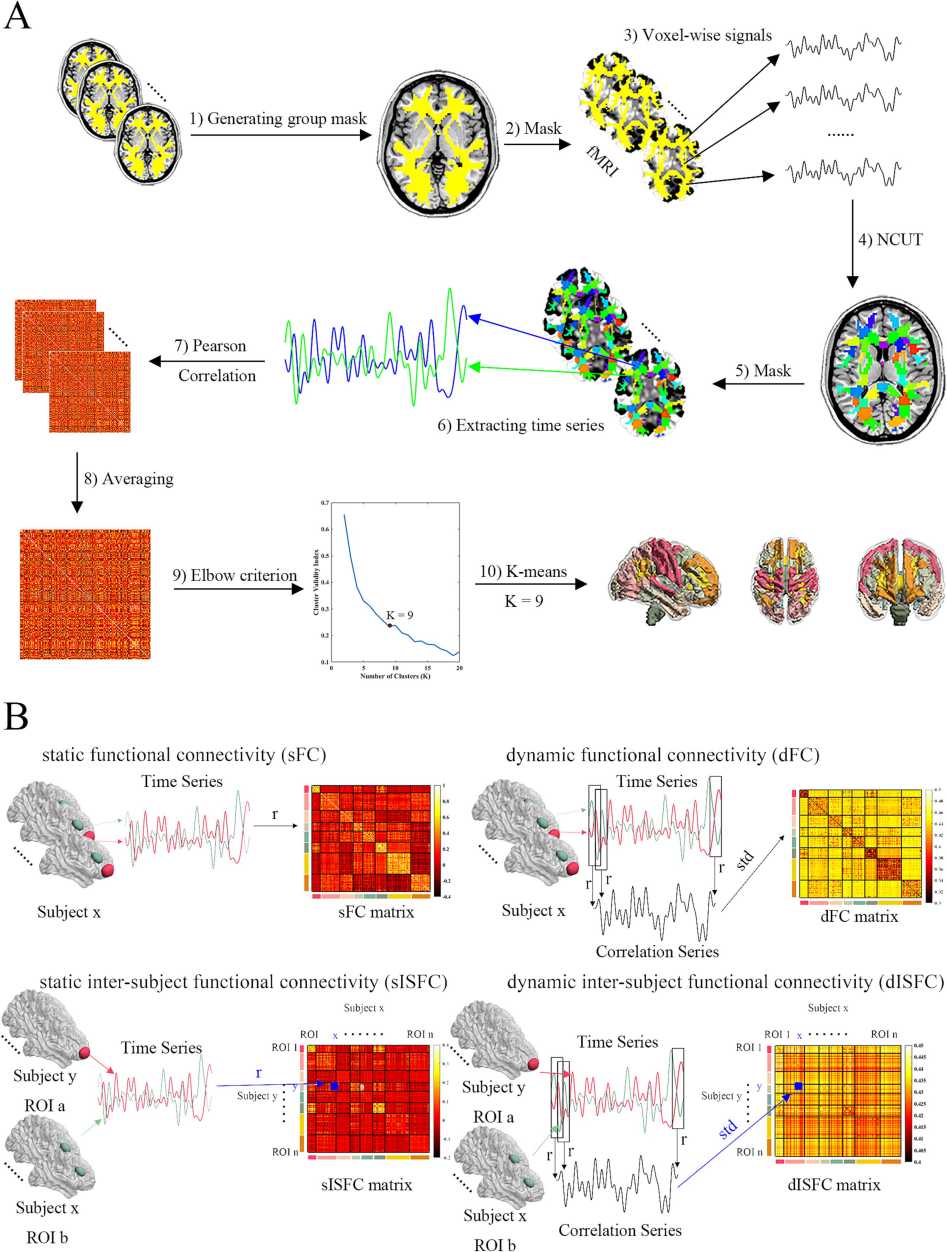
Schema of clustering functional networks and FC measures analysis. **(A)** ROI-level and network-level functional templates were clustered based on normalized cut spectral clustering (NCUT) and K-means in WM. The processing steps were: (1) Group WM mask was generated based on all subjects’ WM masks. (2) All fMRI data were masked by the group WM mask. (3) Voxels-wise signals were extracted from masked fMRI data. (4) 200 ROIs functional template was clustered by NCUT using voxel-wise signals. (5) All fMRI data were masked by ROI-level template. (6) ROI-level signals were extracted from fMRI data after being masked. (7) Every subject’s FC matrices were calculated by Pearson correlation coefficient. (8) These matrices were transformed to z matrices based on Fisher’s z. And they were averaged as group z matrix. Then group FC matrix was converted back from group z matrix. (9) Elbow criterion was used to decide the number of clusters (networks). The cluster validity index was the ratio between within-cluster to between-cluster distance. 9 clusters were determined as the results shown. (10) 9 WM networks were clustered by K-means. **(B)** FC methods. sFC was calculated by Pearson correlation coefficient. dFC was calculated by slice window method. Standard deviation was determined as dFC index. sISFC matrix was calculated based on Pearson correlation coefficient between ROI a and ROI b from different subjects. sISFC-z matrix was calculated by Fisher’s z transform. Group sISFC-z value between ROI a and ROI b was calculated by averaging upper triangular (UT) and lower triangular (LT) of the sISFC-z matrix. Group sISFC value was transformed back from z to r. dISFC was calculated using the slice window method between ROI a and ROI b from different subjects. dISFC index was also the standard deviation. UT and LT of dISFC matrix were averaged as the group dISFC value between ROI a and ROI b.

Group-level structural GM and WM masks were calculated separately by MATLAB scripts. First, spatially normalized GM maps or WM maps were averaged from all subjects (Figure 1A, step 1). Then, the subcortical and cerebellum were removed based on the Harvard-Oxford atlas and Buckner atlas. Only values greater than 0.6 were identified as WM. The voxels were recognized as GM if they were not WM and their values were higher than 0.2. If less than 80% of them from all subjects was not a number based on obtained GM and WM masks, the voxels were removed.

Since the brain function is based on the cooperation of many regions whose voxels may be adjacent in space, normalized cut spectral clustering (NCUT) was determined to generate ROIs by its spatial constraint (Craddock et al., 2012). Because WM had no functional template and we wanted to keep the processing steps similar between GM and WM, we clustered functional templates with 200 ROIs as Craddock and colleagues recommended for GM and WM by using preprocessed REST1 images. As the processing steps to perform clustering for GM and WM were similar, we have only described it for the WM clustering. Voxel-level signals were extracted from REST1 fMRI data using the group-level WM mask (Figure 1A, steps 2, 3). Then, functional template with 200 ROIs was obtained based on NCUT by using voxel-level signals (Figure 1A, step 4). To detect FC on a larger scale compared with ROI-level, we also clustered network-level functional templates for both GM and WM. Similar to the processing for extracting voxel-wise signals, we got ROI-level signals from REST1 images by averaging all voxel time series in the ROIs based on the above template (Figure 1A, steps 5, 6). sFC matrices were calculated by the Pearson correlation coefficient of ROI-level signals (Figure 1A, step 7). We transformed sFC matrices from r to z by using the Fisher’s z transform. They were then averaged to generate the group-level z matrix. The group-level matrix was converted back to r (Figure 1A, step 8). Then, the group-level connectivity matrix was as input of K-means analysis to cluster functional networks from 2 to 20. Based on the elbow criterion of the cluster validity index that was calculated by the ratio between within-cluster to between-cluster distance and the distance was decided by Squared Euclidean distance (Allen et al., 2014). We iterated 100 times to evaluate the relationships between the number of functional networks and the elbow criterion. By drawing the relationship curve figures, 8 GM functional networks and 9 WM functional networks were finally determined because they have more clear “turning point” than other network numbers (Figure 1A, steps 9, 10; Supplementary Figure S1). Because the serial numbers of ROIs in a network were not continuous, we reorganized ROIs based on the networks of GM or WM.

### 2.4. Static FC and static inter-subject FC

Voxel-wise time series were averaged in the ROIs to generate ROI time series for each subject. sFC matrices for all ROI pairs across all subjects were calculated by using Pearson correlation coefficients in MOVIE and REST images (Figure 1B). Fisher’ z transform was performed on all subjects’ sFC matrices and above transformed matrices were averaged as the group-level sFC matrix. The z values of group-level sFC matrix were transformed back to r for more intuitive exhibiting the degree of correlation.

Similar to sFC, the static inter-subject FC (sISFC) was calculated by the Pearson correlation coefficient based on ROI signals. The difference was that sFC was evaluated in the same subject but the sISFC was calculated between different subjects. Since the matrices of sISFC were not symmetrical for each ROI pair and the diagonal values were calculated in the same subject, we averaged each matrix of sISFC excluding the diagonal elements. Specifically, the sISFC between {Subject x – ROI a} and {Subject y – ROI b} is different from the sISFC between {Subject x – ROI b} and {Subject y – ROI a}. Even though both presented the sISFC between ROI a and ROI b. Therefore, we first performed the Fisher’s z analysis to transform r to z values for the ROI a and ROI b sISFC matrix generating sISFC-z matrices. Then, upper angular and lower angular values within the sISFC-z matrix were averaged as the z value of these two ROIs, which meant that the z transformed sISFC matrix was averaged except for its diagonal because the diagonal values were the (intra-subject) sFC. After averaging, the z values were transformed back to r values as the group-level sISFC to show correlation level (Figure 1B). We performed the same steps above for all ROIs to obtain the group-level sISFC matrix.

### 2.5. Dynamic FC and dynamic inter-subject FC

Sliding window analysis has been commonly used to estimate the dynamic FC (dFC; Hutchison et al., 2013; Di and Biswal, 2020; Zhang et al., 2021). Previous studies have indicated higher reliability when the sliding window size was around 30 s in calculating dFC (Hutchison et al., 2013; Allen et al., 2014; Leonardi and Van De Ville, 2015). Therefore, the current study used a sliding window size of 30 time points (30 s), and a time step of 1 time point (1 s). ROI time series were extracted from all subjects by averaging all voxels time series in the ROI from the same subject. Pearson correlation analysis was performed on each sliding window between different ROI time series. Thus, there were a series of correlation coefficients across each pair of ROIs. We calculated the standard deviation of these correlation coefficient series to generate a dFC matrix for each subject (Figure 1B). Group-level dFC was calculated by averaging all subjects’ dFC matrices.

Dynamic inter-subject FC (dISFC) was also computed to detect the dynamic property across different subjects. ROI signals were extracted by averaging all voxels signals in the same ROIs. dISFC was then calculated using different subjects’ ROI signals based on the sliding window with the same parameters described in dFC. As with sISFC, the matrices of dISFC were not symmetric. Therefore, we averaged the upper angular and lower angular of all ROI pairs dISFC matrices as the values of the group-level dISFC matrix (Figure 1B).

### 2.6. Movie-watching vs. resting-state conditions

To estimate the sFC and dFC differences between the movie-watching and the resting-state conditions, MOVIE vs. REST matrices were calculated by subtracting REST matrices from the corresponding MOVIE matrices. The pair-T test was performed on pairs of ROI or network between the resting-state and the movie conditions to determine which ROIs or networks had significant differences between two conditions. For any pair of ROIs, there were 172 (number of subjects) correlation coefficients. Thus, the inputs of pair-T test were the vector of 172 sFC or dFC in the naturalistic condition and the vector of 172 sFC or dFC in the resting-state condition for each pair of ROIs. Fisher’s z transform was performed on the correlation coefficients of sFC before statistics. The above resulting matrices were shown as reverted z to r. Bonferroni correction was performed to estimate the significant differences with *p* < 0.05/*n* (*n* was the number of ROI-pairs or network-pairs).

### 2.7. Movie-evoked FC

An earlier study demonstrated that FC of the movie-watching contained the intrinsic FC in addition to the movie-evoked FC (Lynch et al., 2018). To demonstrate the influence of movie contents, we performed the statistic method of subject-wise bootstrapping (SWB). It has been shown that when using SWB, the false positive rate was the lowest compared with other nonparametric approaches (i.e., element-wise bootstrapping, subject-wise permutation, and element-wise permutation) and student’s *t*-test (Chen et al., 2016). Another reason for not using the student’s t-test is that most of the ISFC values are dependent, violating the principle of independence needed for *t*-test.

We also performed SWB for ISFC. Since the ISC is calculated between pairs of subjects in the same ROI or network, it results in a symmetrical ISC matrix. Thus, Chen and colleagues had only focused on the lower triangles. However, when the ISFC was calculated using different ROIs, the matrix of ISFC was not symmetrical. In other words, ISFC contains ISC (Simony et al., 2016). To solve the asymmetrical matrix of ISFC, we calculated centrality-based measure on both the lower and upper triangles of ISFC matrices without using the diagonal elements of the matrices. Instead of the mean, we performed median as the chosen centrality measure of ISFC to prevent the back-and-forth transformation processing. For example, if using mean value, we should first performed the Fisher’s z transform for correlation coefficients, then average above z values, and further converted the z values back to correlation coefficients (r). Another reason is that the median is less sensitive to biases than the mean when data are not represent normal Gaussian distribution (Chen et al., 2016). The median of the lower and upper triangles of the ISFC matrix was calculated as the statistical parameter from all subjects of each ROI or network. Specifically, SWB was performed as following: (1) The median value of the ISFC matrix was calculated as the raw median value. (2) ISFC values were selected randomly to raw ISFC matrix size as bootstrapping ISFC matrix, then the bootstrapping median value was calculated based on this bootstrapping ISFC matrix. (3) If the bootstrapping median value was higher (lower) than raw median value, the number increases by one. (4) Bootstrapping was performed using 5,000 repetitions from step (2) to step (3). Then *p*-value was calculated based on the number divided by 5,000. (5) Multiple comparison correction was performed by Bonferroni (*p* < 0.05/*n*). (6) All these steps were performed for each ROI or network within GM, WM, and GM-WM. Only the ROI (or network) pairs that passed the multiple comparison correlation were shown.

### 2.8. Reliability

To estimate the reliability of results, the intraclass correlation coefficient (ICC) was performed on the FC or ISFC between Day1 and Day2 (Müller and Büttner, 1994). Specifically, ICC was computed as:


(1) $$ICC=\frac{BMS-WMS}{BMS+\left(k-1\right)WMS}\;$$

where BMS is the between-subjects mean squared variance, WMS is the within-subjects mean squared variance, and k is the repetition number of scans for each participant (k = 2). The reliability of FC and ISFC were calculated as follows: (1) sFC was calculated for all ROI pairs across all subjects separate on Day1 and Day2, resulting in 172 (number of subjects) correlation coefficients for each ROI pair on Day1 or Day2. For each pair of ROIs, both Day1 and Day2 have the vectors of length 172. sFC ICC matrix was calculated based on the vectors between Day1 and Day2 across all ROI pairs. For dFC ICC, we used standard deviation to calculate ICC. Finally, sFC ICC and dFC ICC in GM, WM, and GM-WM were estimated based on these steps above within network- and ROI-levels. (2) For sISFC, we first calculated Pearson correlation coefficients between subjects for each ROI pairs, resulting in the sISFC matrix for each pair of ROIs. The lower and upper triangles of sISFC matrix (excluding diagonal elements) were reshaped to a vector (length is 172 × 172 − 172 = 29,412). After arranging the vectors for both Day1 and Day2, sISFC ICC was calculated using the vectors of Day1 and Day2 for each pair of ROIs. Similar to dFC ICC, we analyzed the dISFC ICC using its standard deviation. These processing steps were performed in GM, WM, and GM-WM at the network- and ROI-levels separately.

After calculating the ICC, permutation analysis of ICC was performed between the movie-watching and the resting-state. The raw difference was calculated by mean ICC value of movie-watching subtracting mean ICC value of resting-state. The permutation was iterated with 5,000 times. In each iteration, ICC matrices between two conditions were shuffled, then separated to two matrices. The random difference was calculated by minus between mean values of these two matrices. The count was added when the raw difference was lower than the random difference. The p value was calculated by the count dividing by the iteration number.

## 3. Results

### 3.1. Clustering GM and WM functional networks

**Figure 2:**
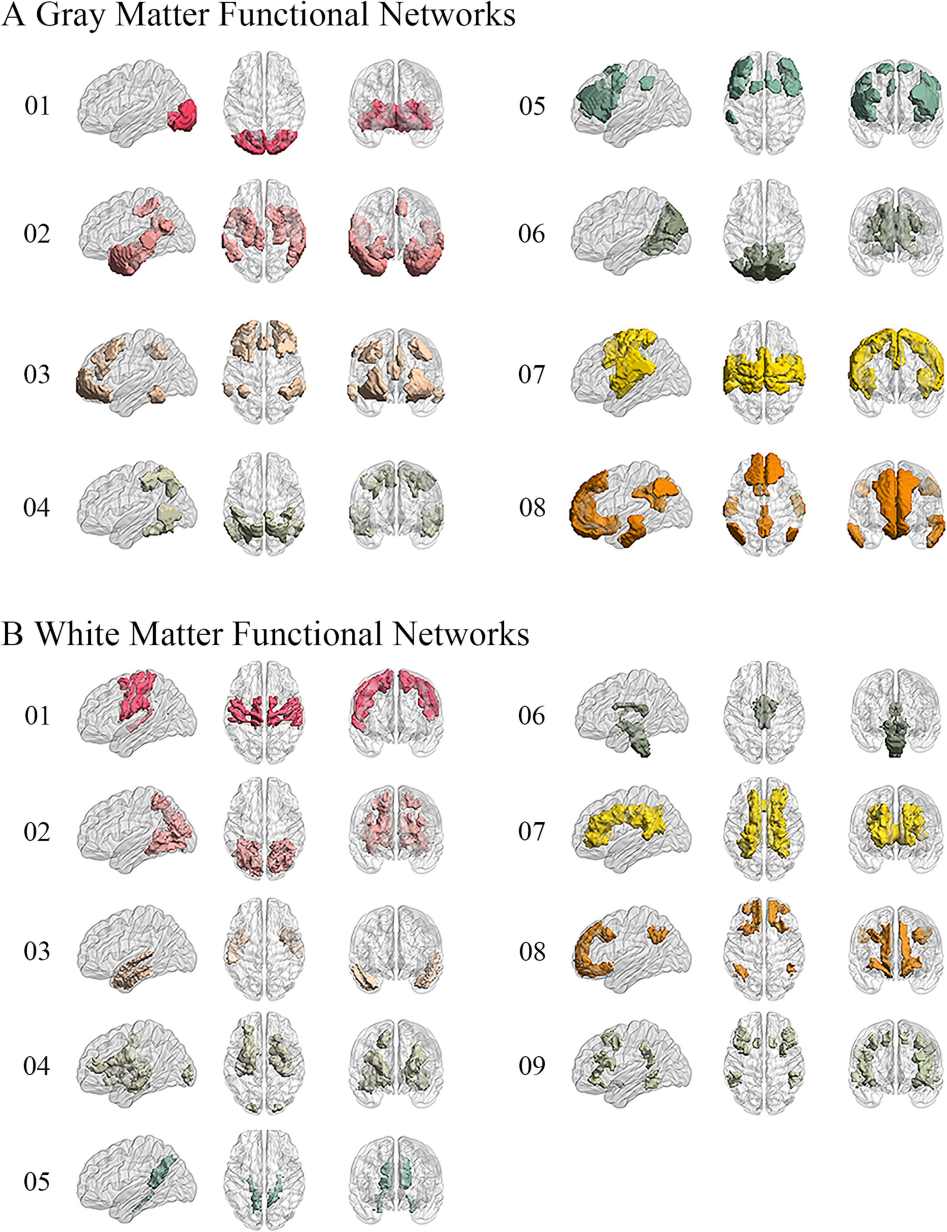
Obtaining 8 GM and 9 WM functional networks. **(A)** GM networks: 01. Lateral visual network (LVN); 02. Limbic network (LIMB); 03. Frontoparietal network (FPN); 04. Dorsal attention network (DAN); 05. Ventral attention network (VAN); 06. Medial visual network (MVN); 07. Sensorimotor network (SMN); 08. Default mode network (DMN). **(B)** WM networks: 01. Sensorimotor network in WM (SMN-WM); 02. Occipital network in WM (ON-WM); 03. Superior temporal network in WM (STN-WM); 04. Anterior corona radiata network in WM (ACRN-WM); 05. Posterior corona radiata network in WM (PCRN-WM); 06. Inferior corticospinal network in WM (ICN-WM); 07. Deep network in WM (DN-WM); 08. Orbitofrontal network in WM (OFN-WM); 09. Frontoparietal network in WM (FPN-WM).

We performed the elbow criterion of the cluster validity index to estimate the numbers of functional networks in GM and WM. We found that the optimal numbers of functional networks in GM and WM were 8 and 9, respectively. These functional networks within GM were: lateral visual network (LVN), limbic network (LIMB), frontoparietal network (FPN), dorsal attention network (DAN), ventral attention network (VAN), medial visual network (MVN), sensorimotor network (SMN), and default mode network (DMN). These results have been labeled and shown in Figure 2. The primary networks contained LVN, MVN, and SMN. The high-level networks contained LIMB, FPN, DAN, VAN, and DMN. The functional networks in WM were: sensorimotor network (SMN-WM), occipital network (ON-WM), superior temporal network (STN-WM), anterior corona radiata network (ACRN-WM), posterior corona radiata network (PCRN-WM), inferior corticospinal network (ICN-WM), deep network (DN-WM), orbitofrontal network (OFN-WM), frontoparietal network (FPN-WM; Figure 2). The superficial layers contained SMN-, ON-, STN-, OFN-, and FPN-WMs. The middle layers contained ACRN- and PCRN-WMs. The deep layer contained DN-WM. The ICN-WM across superficial and middle layers.

To compare between the results of clustering and traditional networks, we decided the Yeo7 GM networks as the traditional GM networks and the Peer12 WM networks as the traditional WM networks. The Dice Coefficient was determined as the similarity algorithm. As the result shown, somatomotor (Sensorimotor), dorsal attention, ventral attention, limbic, frontoparietal, and default mode networks indicated the high connection between the Yeo’s networks and our networks. The visual network of Yeo was separate to two networks, which were lateral visual network and medial visual network. For WM networks, some of our WM networks integrated Peer’s WM networks. To be specific, sensorimotor network contained semsorimotor superficial white-matter system and dorsal frontoparietal tracts; occipital network contained visual superficial white-matter system and inferior longitudinal fasciculus system; superior temporal network was similar to uncinate and middle temporal lobe tracts; anterior corona radiata network was similar to ventral frontoparietal tracts; posterior corona radiata network was similar to cingulum and associated tracts; inferior corticospinal network contained inferior corticospinal tract and posterior cerebellar tracts; deep network contained superior longitudinal fasciculus system; orbitofrontal network was similar to forceps minor system; frontoparietal network was similar to ventral frontoparietal tracts (Supplementary Figure S2).

### 3.2. FC and ISFC in GM

**Figure 3:**
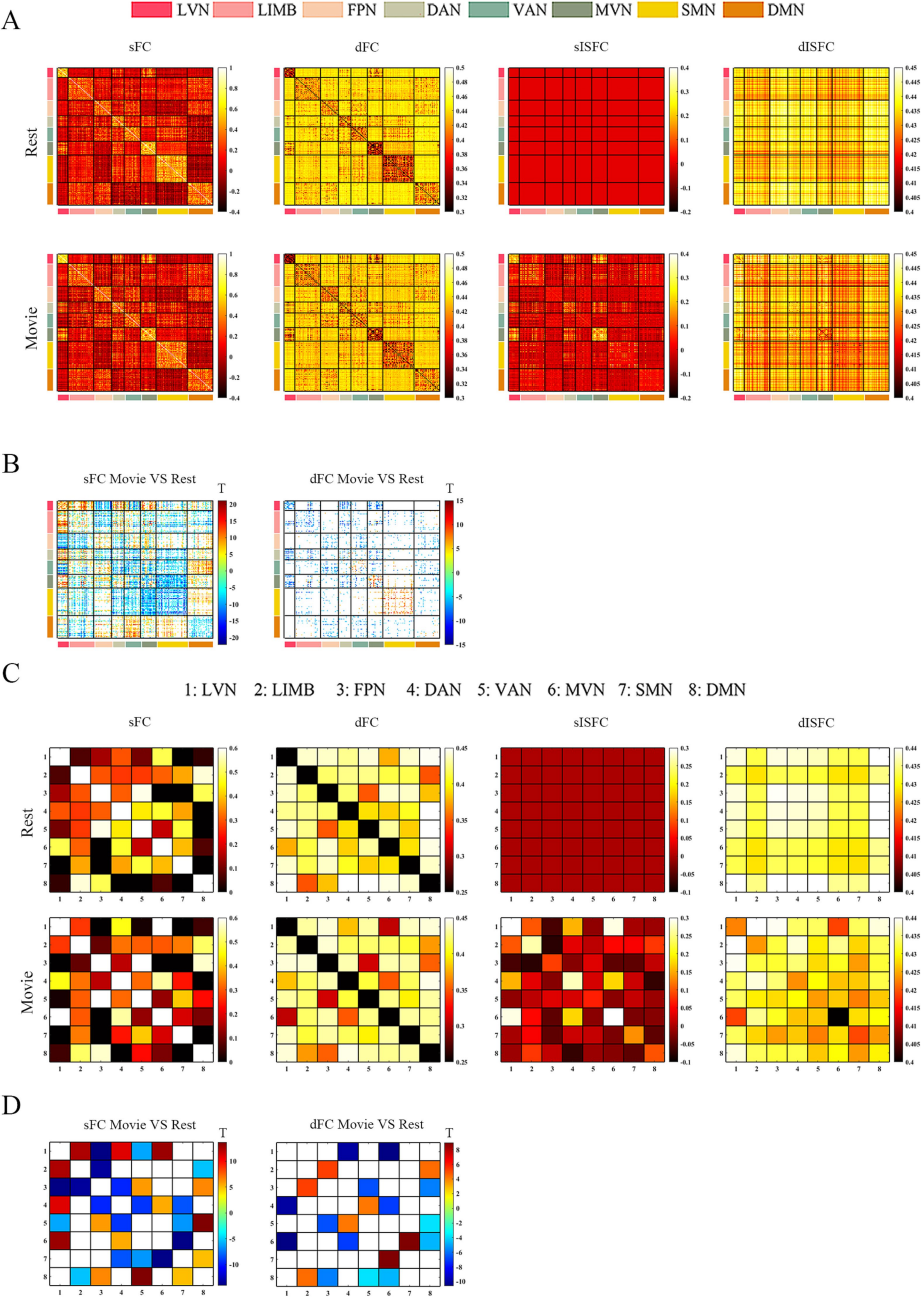
ROI- and network-levels FC and ISFC matrices in GM. **(A)** ROI-level sFC, dFC, sISFC, and dISFC matrices were calculated separately in the resting-state and the movie-watching. **(B)** sFC and dFC Movie vs. Rest matrices were calculated by subtracting REST matrices from the corresponding MOVIE matrices. They were performed the pair-T test and were corrected by Bonferroni. ROIs that did not pass the Bonferroni were set blank. **(C)** Networks-level sFC, dFC, sISFC, and dISFC matrices were calculated separately in the resting-state and the movie-watching. **(D)** The processing steps within network-level sFC and dFC Movie vs. Rest matrices were similar to ROI-level. LVN, Lateral visual network; LIMB, Limbic network; FPN, Frontoparietal network; DAN, Dorsal attention network; VAN, Ventral attention network; MVN, Medial visual network; SMN, Sensorimotor network; DMN, Default mode network.

At the ROI-level and network-level analyses of GM regions, the resting-state and the movie-watching had similar functional patterns of sFC (Figures 3A,C). sFC demonstrated higher FC strengths within intra-networks than them within inter-networks (Figure 3A). We analyzed the differences of sFC between movie and rest conditions, and found the positive and negative values both existed in the difference matrix of sFC (Figure 3B). To better discuss the FC within networks, we calculated number of elements, number of passed correction elements, number of positive elements, number of negative elements, and mean values within networks. More than half of the elements relating to LVN, MVN, and SMN passed significance based on Bonferroni correction. The above three networks had different performances of sFC during the resting-state and the movie-watching. All significant ROI pairs relating to LVN had higher sFC values in the naturalistic viewing, indicating the movie-watching heightened sFC relating to LVN. On the contrary, for significant ROI pairs in the SMN, the movie-watching condition showed weaker sFC than that during the resting-state. For MVN, most ROI pairs exhibited enhanced sFC, but 19% of ROIs showed negative values. The averaged sFC values within the various networks ranged between −0.077 and 0.07 (Table 3). For the sFC Movie vs. Rest matrix at the network-level, primary networks had different performances. Specifically, in the movie-watching, sFC between LVN and MVN was higher than that in the resting state. But the sFC between MVN and SMN was stronger under the resting-state than under the naturalistic condition. The conditions had no significant difference between LVN and SMN. Between primary and high-level networks, sFC values between LVN and LIMV and between LVN and DAN were improved by the movie stimuli. However, sFC values were lower in the movie-watching condition than in the resting-state between LVN and FPN, between LVN and VAN, as well as between SMN and DAN. For the high-level networks, sFC between VAN and DMN was higher in the movie condition than that during the resting-state. However, sFC became weaker under the movie stimuli between LIMB and FPN, between FPN and DAN, as well as between DAN and VAN (Figure 3D).

**Table 3:** sFC and dFC in GM networks.

Networks	NoE	NoPE (NoPE/NoE)		NoP (NoP/NoPE)		NoN (NoN/NoPE)		Mean	
		sFC	dFC	sFC	dFC	sFC	dFC	sFC	dFC
LVN	225	138 (61%)	68 (30%)	138 (100%)	0 (0%)	0 (0%)	68 (100%)	0.070	−0.017
LIMB	1,089	300 (28%)	120 (11%)	166 (55%)	0 (0%)	134 (45%)	120 (100%)	0.004	−0.009
FPN	529	134 (25%)	70 (13%)	116 (87%)	0 (0%)	18 (13%)	70 (100%)	0.027	−0.011
DAN	256	72 (28%)	46 (18%)	30 (42%)	8 (17%)	42 (58%)	38 (83%)	−0.014	−0.007
VAN	441	196 (44%)	50 (11%)	14 (7%)	34 (68%)	182 (93%)	16 (32%)	−0.051	0.001
MVN	400	210 (53%)	106 (27%)	40 (19%)	80 (75%)	170 (81%)	26 (25%)	−0.051	0.010
SMN	1,600	798 (50%)	242 (15%)	0 (0%)	240 (99%)	798 (100%)	2 (1%)	−0.077	0.010
DMN	1,024	292 (29%)	24 (2%)	4 (1%)	10 (42%)	288 (99%)	14 (58%)	−0.046	0

NoE, number of elements; NoPE, number of passed correction elements; NoP, number of positive elements from correction elements; NoN, number of negative elements from correction elements. LVN, lateral visual network in GM; LIMB, limbic network in GM; FPN, frontoparietal network in GM; DAN, dorsal attention network in GM; VAN, ventral attention network in GM; MVN, medial visual network in GM; SMN, sensorimotor network in GM; DMN, default mode network in GM.

The dFC patterns were similar in general between the resting-sate and the movie-watching at the ROI-level and network-level (Figures 3A,C). dFC showed weaker connectivity fluctuations in intra-networks than those in inter-networks (Figure 3A). We analyzed the differences of dFC between Movie and Rest conditions, and the positive and negative values were found in the difference matrix of dFC (Figure 3B). The top 3 GM networks were LVN, MVN, and DAN, which had most elements that passed multiple correction. Specifically, 30% of ROIs in the LVN have passed correction. It was found that all of them were weaker under the movie-watching than those in the resting-state. 27% of ROIs in the MVN demonstrated the significance. 75% of these ROIs’ values were positive and 25% of them were negative. 18% of elements in the DAN have passed the correction. 17% of them were positive and 83% of them were negative. The range of averaged dFC values was from −0.017 to 0.01 (Table 3). As the dFC Movie vs. Rest matrix at the network-level, dFC between LVN and MVN became lower in movie-watching than in the resting-state. But dFC between MVN and SMN was higher in the naturalistic condition than that in the resting-state. Between primary and high-level networks, dFC showed lower values under the movie-watching than them in the resting-state between LVN and DAN as well as between DAN and MVN. Within high-level networks, dFC values were lower in the movie condition than them during the resting-state between FPN and VAN. But, the movie stimuli improved dFC values between LIMB and FPN, between DAN and VAN, and between LIMB and DMN (Figure 3D).

We did not find the specific functional pattern of sISFC and dISFC in the resting-state at the ROI-level. Under the movie-watching condition, compared to other functional networks, the sISFC values relating to LVN and MVN exhibited enhancement. However, dISFC did not show clear functional patterns (Figure 3A). At the network-level, compared with the naturalistic condition, the resting-state did not show the clear sISFC and dISFC patterns. sISFC values of LVN, MVN, as well as between LVN and MVN were improved by the movie-watching condition. Compared with LVN, the enhancements of dISFC in MVN and between LVN and MVN were more evident (Figure 3C).

### 3.3. FC and ISFC in WM

**Figure 4:**
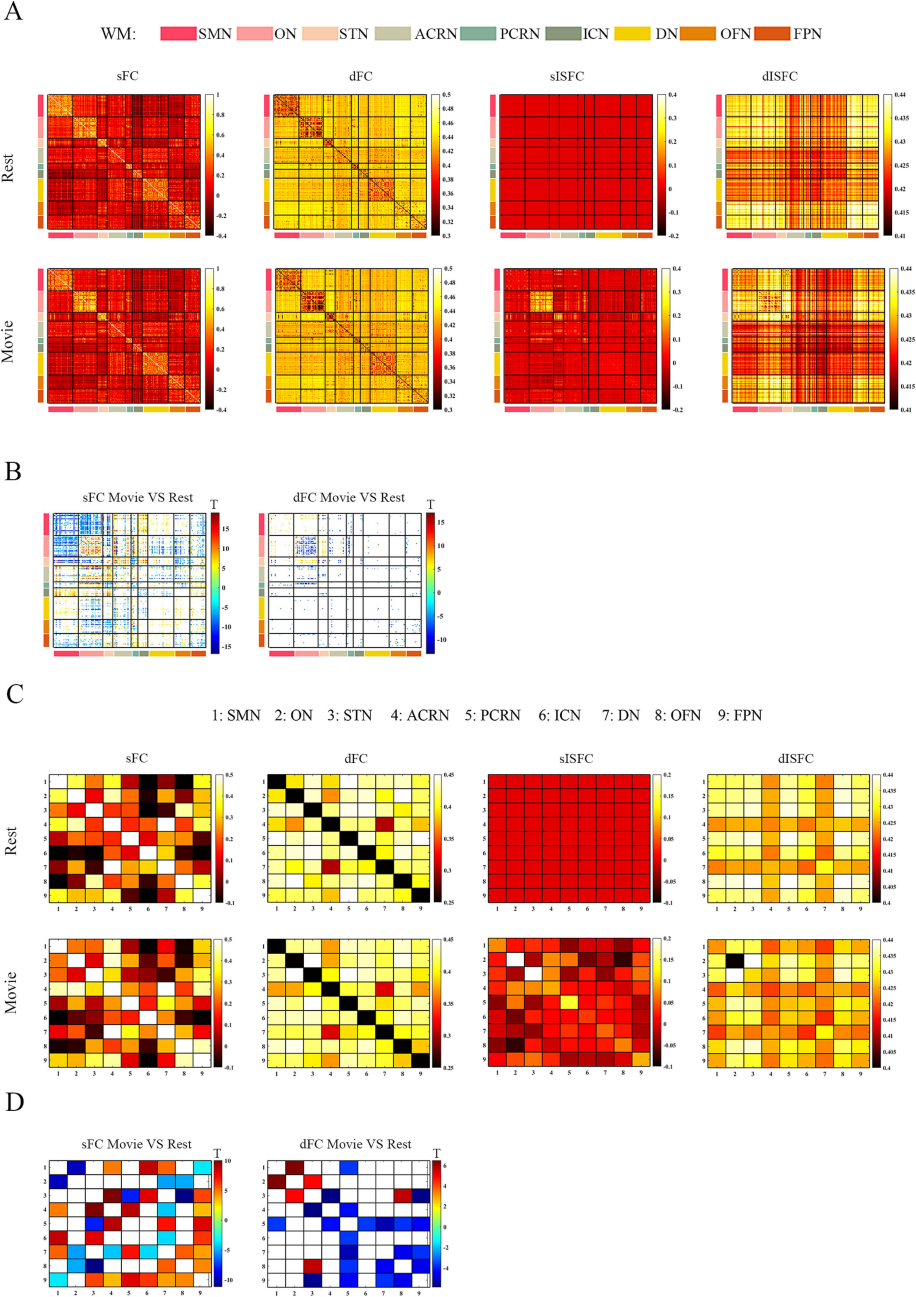
ROI- and network-levels FC and ISFC matrices in WM. **(A)** ROI-level sFC, dFC, sISFC, and dISFC matrices were calculated separately in the resting-state and the movie-watching. **(B)** sFC and dFC Movie vs. Rest matrices were calculated by subtracting REST matrices from the corresponding MOVIE matrices. They were performed the pair-T test and were corrected by Bonferroni. ROIs that did not pass the Bonferroni were set blank. **(C)** Networks-level sFC, dFC, sISFC, and dISFC matrices were calculated separately in the resting-state and the movie-watching. **(D)** The processing steps within Network-level sFC and dFC Movie vs. Rest matrices were similar to ROI-level. SMN, Sensorimotor network; ON, Occipital network; STN, Superior temporal network; ACRN, Anterior corona radiata network; PCRN, Posterior corona radiata network; ICN, Inferior corticospinal network; DN, Deep network; OFN, Orbitofrontal network; FPN, Frontoparietal network.

As shown in the sFC matrices at the ROI-level and network-level, the movie-watching did not modify the functional patterns compared with the resting-state (Figures 4A,C). About the Movie vs. Rest matrix of sFC, the naturalistic condition increased FC within some networks, but the opposite situation also existed in some networks (Figure 4B). Specifically, about 30% of the ROIs passed the multiple comparison correction in the SMN-WM, and most (99%) of these ROIs were negative values. 33% of ROIs demonstrated significance within ON-WM, and 77% of them were positive values (Table 4). As the sFC Movie vs. Rest matrix at the network-level, sFC values between SMN- and ON-WMs and between STN- and OFN-WMs were lower in the naturalistic condition than them in the resting-state within the superficial layer. However, sFC between STN- and FPN-WMs was higher in the movie-watching than that during the resting-state within the superficial layer. As the WM network crosses superficial and middle layers, ICN-WM showed high sFC values with superficial networks, including SMN-, STN-, and FPN-WMs. As layers between superficial and middle, the sFC between STN- and PCRN-WMs was weak in the naturalistic condition. However, the sFC values between STN- and ACRN-WMs, and between PCRN- and FPN-WMs became strong from the resting-state to the movie condition. For layers between superficial and deep, the sFC between ON- and DN-WMs was lower in the movie-watching while sFC values between SMN- and DN-WMs, and between DN- and OFN-WMs were higher in the movie-stimuli than them during the resting-state. The sFC between ACRN- and PCRN-WMs was improved by the movie-watching in the middle layer. Between middle and deep layers, the sFC between PCRN- and DN-WMs was higher in the movie condition than that during the resting-state (Figure 4D).

**Table 4:** sFC and dFC in WM networks.

Networks	NoE	NoPE (NoPE/NoE)		NoP (NoP/NoPE)		NoN (NoN/NoPE)		Mean	
		sFC	dFC	sFC	dFC	sFC	dFC	sFC	dFC
SMN-WM	1,156	352 (30%)	42 (4%)	2 (1%)	40 (95%)	350 (99%)	2 (5%)	−0.067	0.005
ON-WM	1,024	342 (33%)	224 (22%)	262 (77%)	38 (17%)	80 (23%)	186 (83%)	0.028	−0.008
STN-WM	169	16 (9%)	6 (4%)	14 (88%)	0 (0%)	2 (12%)	6 (100%)	0.016	−0.007
ACRN-WM	576	102 (18%)	2 (0.4%)	68 (67%)	0 (0%)	34 (33%)	2 (100%)	0.010	−0.004
PCRN-WM	81	12 (15%)	0 (0%)	0 (0%)	0	12 (100%)	0	−0.034	−0.002
ICN-WM	169	26 (15%)	0 (0%)	0 (0%)	0	26 (100%)	0	−0.055	0.001
DN-WM	1,156	20 (2%)	2 (0.2%)	18 (90%)	0 (0%)	2 (10%)	2 (100%)	−0.001	−0.002
OFN-WM	441	22 (5%)	12 (3%)	14 (64%)	0 (0%)	8 (36%)	12 (100%)	0.010	−0.008
FPN-WM	400	52 (13%)	2 (0.5%)	2 (4%)	0 (0%)	50 (96%)	2 (100%)	−0.022	−0.003

NoE, number of elements; NoPE, number of passed correction elements; NoP, number of positive elements from correction elements; NoN, number of negative elements from correction elements; SMN-WM, sensorimotor network in WM; ON-WM, occipital network in WM; STN-WM, superior temporal network in WM; ACRN-WM, anterior corona radiata network in WM; PCRN-WM, posterior corona radiata network in WM; ICN-WM, inferior corticospinal network in WM; DN-WM, deep network in WM; OFN-WM, orbitofrontal network in WM; FPN-WM, frontoparietal network in WM.

The dFC patterns under the movie-watching condition were similar to the corresponding values in the resting-state at the ROI-level and network-level (Figures 4A,C). For the dFC Movie vs. Rest matrix at the ROI-level, 22% of ROIs in the ON-WM demonstrated the significance and 83% of them were negative. Only a small fraction of elements (4%) passed multiple comparison correction in the SMN-WM (Table 4). For the dFC Movie vs. Rest matrix at the network-level, dFC between STN- and FPN-WMs of the superficial layers was lower during the movie-watching than that in the resting-state. But dFC values between SMN- and ON-WMs, and between STN- and OFN-WMs were higher in the movie-stimuli than them during the resting-state. Between superficial and middle layers, dFC between STN- and ACRN-WMs became low from the resting-state to the movie-watching. For layers between middle and deep, the dFC also got low in the movie condition compared with the resting-state between PCRN- and DN-WMs (Figure 4D).

For ISFC matrices at the ROI-level, only the sISFC matrix of the movie-watching demonstrated distinct functional patterns. The sISFC patterns of ON- and STN-WMs were found to be distinct during the movie watching (Figure 4A). For sISFC and dISFC matrices at the network-level, the resting-state did not show clear patterns. The movie stimuli have improved sISFC values of the ON- and STN-WMs. Compared with sISFC matrix, only the ON-WM showed a relatively stable signal due to its low dISFC value during the movie-watching. On the other hand, inter-networks connectivity had relatively fewer sISFC and higher dISFC values compared with the intra-networks (Figure 4C).

### 3.4. FC and ISFC in GM-WM

**Figure 5:**
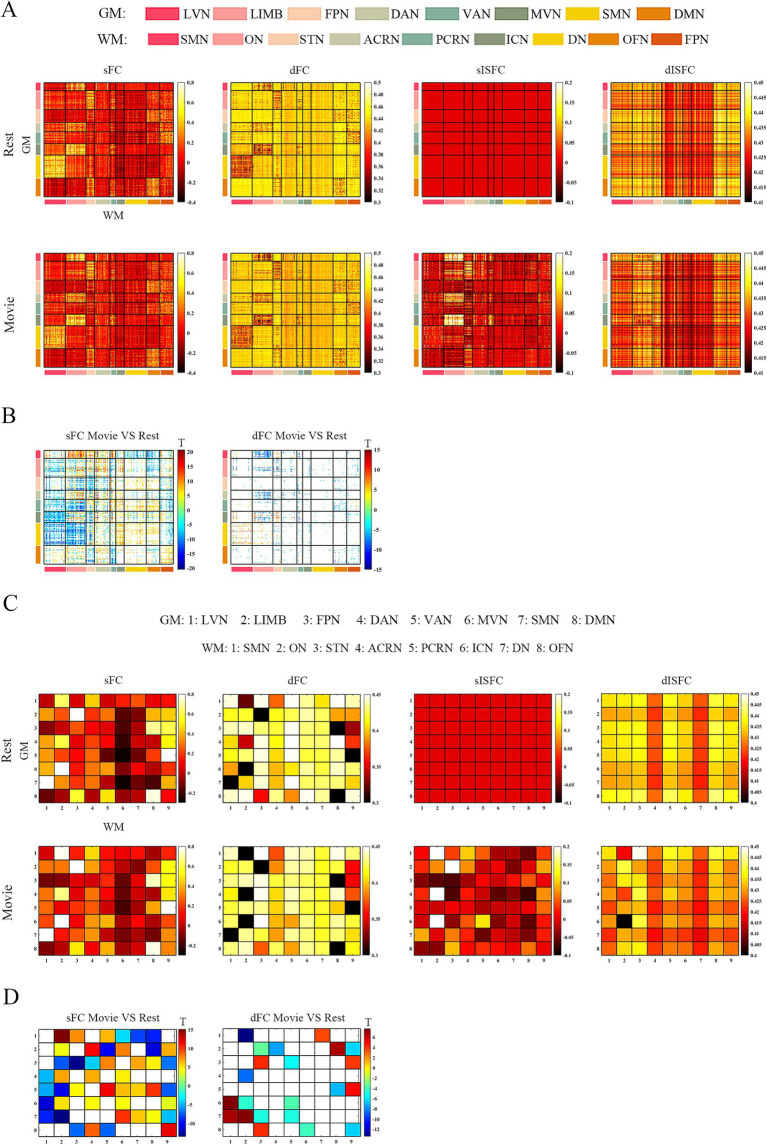
ROI- and network-levels FC and ISFC matrices between GM and WM. **(A)** ROI-level sFC, dFC, sISFC, and dISFC matrices were calculated separately in the resting-state and the movie-watching. **(B)** sFC and dFC Movie vs. Rest matrices were calculated by subtracting REST matrices from the corresponding MOVIE matrices. They were performed the pair-T test and were corrected by Bonferroni. ROIs that did not pass the Bonferroni were set blank. **(C)** Networks-level sFC, dFC, sISFC, and dISFC matrices were calculated separately in the resting-state and the movie-watching. **(D)** The processing steps within Network-level sFC and dFC Movie vs. Rest matrices were similar to ROI-level. GM: LVN, Lateral visual network; LIMB, Limbic network; FPN, Frontoparietal network; DAN, Dorsal attention network; VAN, Ventral attention network; MVN, Medial visual network; SMN, Sensorimotor network; DMN, Default mode network. WM: SMN, Sensorimotor network; ON, Occipital network; STN, Superior temporal network; ACRN, Anterior corona radiata network; PCRN, Posterior corona radiata network; ICN, Inferior corticospinal network; DN, Deep network; OFN, Orbitofrontal network; FPN, Frontoparietal network.

Like GM and WM, the resting-state and the movie-watching had similar sFC patterns at the ROI-level and the network-level (Figures 5A,C). Unlike GM or WM in which intra-network FC values were talked at the ROI-level, we calculated Pearson correlation coefficients between GM and WM networks both in the resting-state and during the movie-watching. In general, the correspondence between GM and WM functional networks were similar, and the only difference was that the GM network corresponding to the PCRN-WM became the MVN under movie-watching condition from DMN during the resting-state. However, as the absolute difference of correlation coefficients between MVN-PCRN (GM-WM networks) and DMN-PCRN was less than 0.01, GM network could be thought as the DMN corresponding to the PCRN-WM (Supplementary Tables S1, S2). For the matrix of sFC Movie vs. Rest at the ROI-level, 40% of elements in SMN-SMN and MVN-ON passed correction. 99% of SMN-SMN values were negative. 47% of MVN-ON values were positive and 53% of them were negative. 32% of elements in LVN-ACRN, DMN-PCRN, and LVN-DN have demonstrated significance. 77% of LVN-ACRN values were positive. 100% of DMN-PCRN and 95% of LVN-DN values were negative (Table 5). Under the movie-watching condition, the sFC matrix showed stronger connectivity for MVN-ON and SMN-SMN. However, we also observed weaker connectivity between GM-WM networks including the LVN-DN, LVN-ICN, VAN-FPN, and DMN-PCRN in the movie condition. LVN-ACRN, LIMB-STN, and DMN-OFN did not show significant differences between the resting-state and the naturalistic conditions (Figure 5D).

**Table 5:** sFC and dFC between GM and WM networks.

Networks (GM-WM)	NoE	NoPE (NoPE/NoE)		NoP (NoP/NoPE)		NoN (NoN/NoPE)		Mean	
		sFC	dFC	sFC	dFC	sFC	dFC	sFC	dFC
SMN-SMN	1,360	546 (40%)	128 (9%)	8 (1%)	117 (91%)	538 (99%)	11 (9%)	−0.014	−0.004
MVN-ON	640	259 (40%)	129 (20%)	121 (47%)	51 (40%)	138 (53%)	78 (60%)	−0.006	−0.005
LIMB-STN	429	117 (27%)	47 (11%)	65 (56%)	7 (15%)	52 (44%)	40 (85%)	−0.027	−0.004
LVN-ACRN	360	115 (32%)	14 (4%)	88 (77%)	5 (36%)	27 (23%)	9 (64%)	0.009	−0.006
DMN-PCRN	288	93 (32%)	9 (3%)	0 (0%)	2 (22%)	93 (100%)	7 (78%)	0.045	−0.008
LVN-ICN	195	26 (13%)	0 (0%)	8 (31%)	0	18 (69%)	0	0.033	−0.006
LVN-DN	510	165 (32%)	0 (0%)	9 (5%)	0	156 (95%)	0	0.038	−0.006
DMN-OFN	672	72 (11%)	26 (4%)	24 (33%)	0 (0%)	48 (67%)	26 (100%)	−0.007	−0.006
VAN-FPN	420	117 (28%)	10 (2%)	10 (9%)	7 (70%)	107 (91%)	3 (30%)	0.051	−0.009

NoE, number of elements; NoPE, number of passed correction elements; NoP, number of positive elements from correction elements; NoN, number of negative elements from correction elements; SMN-SMN, sensorimotor network in GM and sensorimotor network in WM; MVN-ON, medial visual network in GM and occipital network in WM; LIMB-STN, limbic network in GM and superior temporal network in WM; LVN-ACRN, lateral visual network in GM and anterior corona radiata network in WM; DMN-PCRN, default mode network in GM and posterior corona radiata network in WM; LVN-ICN, lateral visual network in GM and inferior corticospinal network in WM; LVN-DN, lateral visual network in GM and deep network in WM; DMN-OFN, default mode network in GM and orbitofrontal network in WM; VAN-FPN, ventral attention network in GM and frontoparietal network in WM.

dFC of the resting-state and movie-watching performed similar functional patterns at the ROI-level and the network-level (Figures 5A,C). As dFC Movie vs. Rest matrices at the ROI-level, MVN-ON demonstrated most correction elements. Still, only 20% of elements passed multiple comparison correction. 40% of their values were positive and 60% of their values were negative. There were too few elements left after correction for other networks between GM and WM (Table 5). For the matrix of dFC Movie vs. Rest at the network-level, MVN-ON, LVN-ACRN, LVN-DN, DMN-OFN and VAN-FPN exhibited similar connectivity patterns with the sFC results. For other networks between GM and WM, dFC of SMN-SMN was higher while LIMB-STN performed weaker dFC value under the naturalistic viewing than them during the resting-state. Furthermore, LVN-ICN and DMN-PCRN did not show significant differences between the resting-state and the movie condition (Figure 5D).

For four matrices of ISFC at the ROI-level, only sISFC matrix in the movie condition showed functional patterns. sISFC values in LVN-ON and MVN-ON were stronger than other networks (Figure 5A). At the network-level, sISFC and dISFC did not denote any clear functional patterns in the resting-state. sISFC values of LVN-ON, DAN-ON, MVN-ON and LIMB-STN were improved by the naturalistic stimuli. For dISFC in movie-watching, only MVN-ON performed weaker signal fluctuation compared with the resting-state (Figure 5C).

### 3.5. Movie-evoked FC

The subject-wise bootstrapping (SWB) was performed to detect the significant activation induced by the movie stimuli. However, we did not observe any ROI or network that exhibited the significant activation in both sISFC and dISFC. We also repeated the analysis steps in the Day2 data and the results were similar to Day1. Though the movie-watching did enhance ISFC of the visual networks for both GM and WM, as the matrices have shown in Figure 5, the improvements were not strong enough to pass the nonparametric approach.

### 3.6. Reliability analysis

In general, the intraclass correlation coefficients (ICCs) in the movie-watching were higher than them during the resting-state in GM, WM, and GM-WM. In detail, sFC and sISFC values in the resting-state were low, and their ICCs were poor. sISFC and dISFC values of visual networks denoted relatively high reliability including the LVN, MVN, and ON-WM than other networks. sISFC and dISFC values between visual networks of GM and WM have also shown strong reliability. These results about visual networks performed similarly at the ROI-level and network-level.

**Figure 6:**
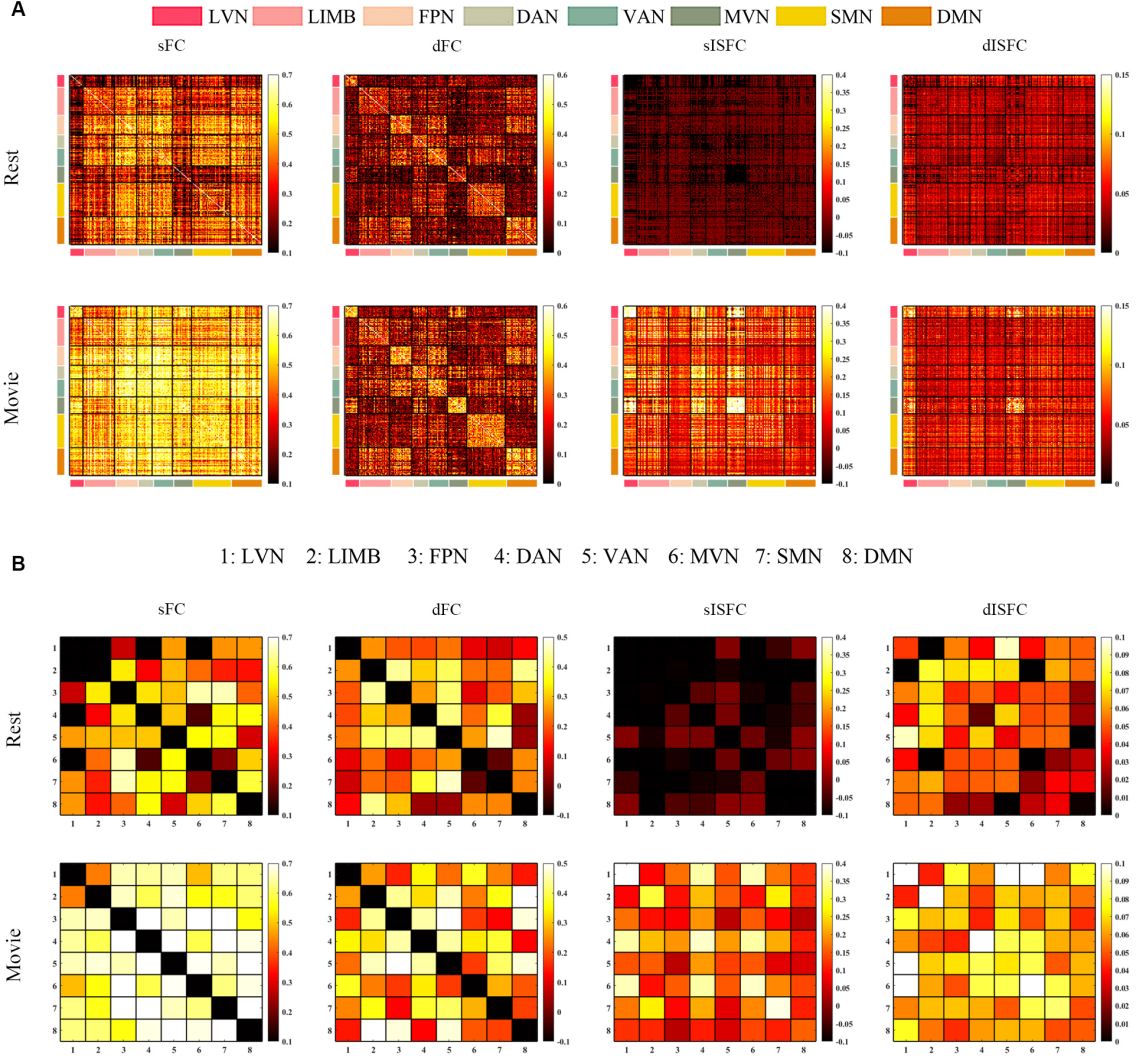
The ICC analysis of FC and ISFC in GM. **(A)** ROI-level sFC, dFC, sISFC, and dISFC ICC matrices were calculated separately in the resting-state and movie-watching. **(B)** Network-level sFC, dFC, sISFC, and dISFC ICC matrices were calculated in the resting-state and the movie-watching separately. LVN, Lateral visual network. LIMB, Limbic network; FPN, Frontoparietal network; DAN, Dorsal attention network; VAN, Ventral attention network; MVN, Medial visual network; SMN, Sensorimotor network; DMN, Default mode network.

Compared with the resting state condition, the sISFC ICC of SMN at network-level under the movie-watching showed relative improvement. At the ROI-level, the sISFC ICC of SMN was smaller than the corresponding value obtained at the network-level. The sISFC ICC of DAN exhibited a similar result between ROI- and network-levels under the movie-watching condition. The network-level dFC ICCs between FPN and VAN and between LIMB and DMN were relatively high, and their ROI-level results had a similar result (Figure 6).

**Figure 7:**
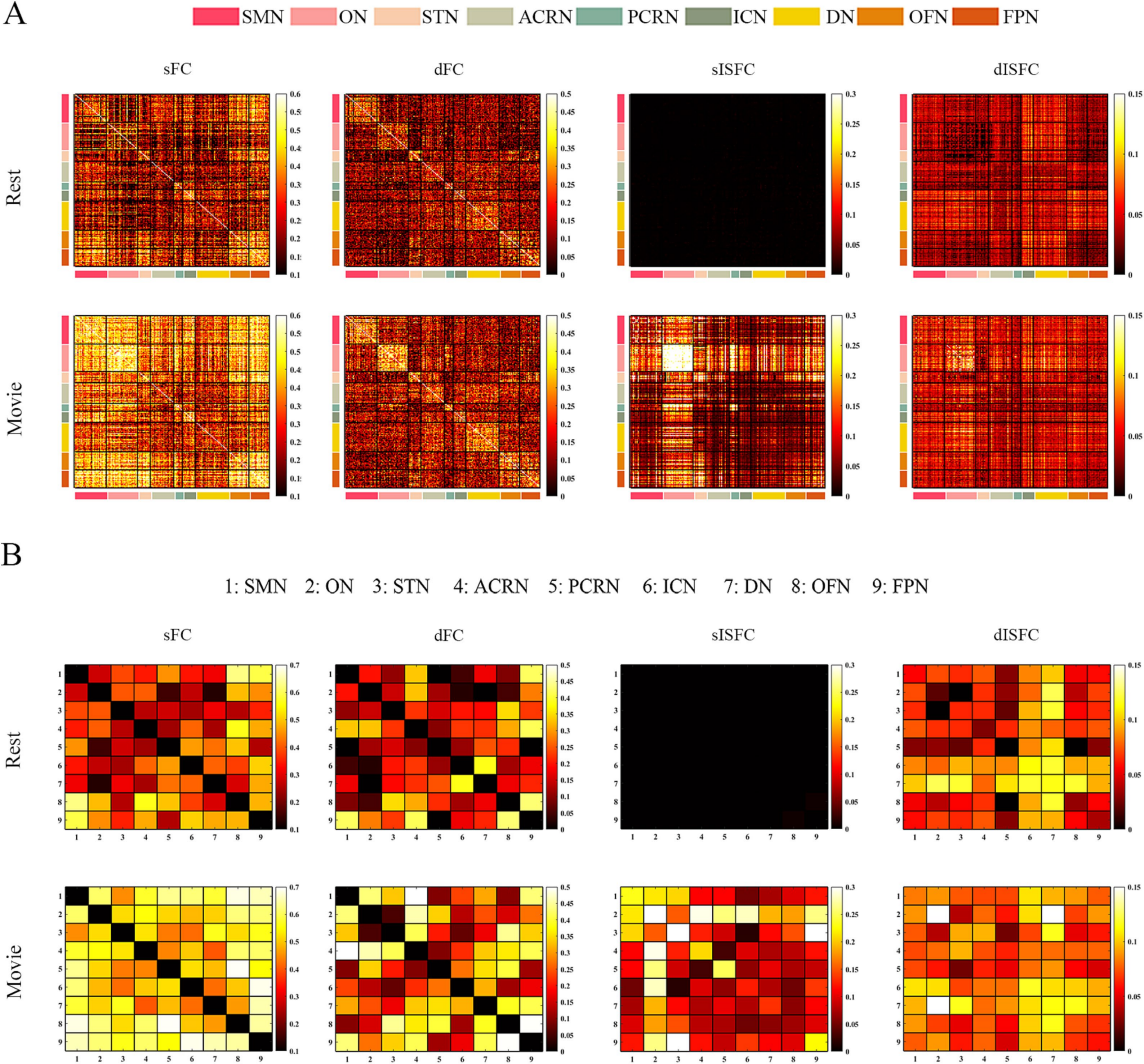
The ICC analysis of FC and ISFC in WM. **(A)** ROI-level sFC, dFC, sISFC, and dISFC ICC matrices were calculated separately in the resting-state and movie-watching. **(B)** Network-level sFC, dFC, sISFC, and dISFC ICC matrices were calculated in the resting-state and the movie-watching separately. SMN, Sensorimotor network; ON, Occipital network; STN, Superior temporal network; ACRN, Anterior corona radiata network; PCRN, Posterior corona radiata network; ICN, Inferior corticospinal network; DN, Deep network; OFN, Orbitofrontal network; FPN, Frontoparietal network.

For WM, the sISFC ICCs within STN-WM, between ON- and ACRN-WMs, and between STN- and FPN-WMs were high in the movie-watching. As the dFC, the ICCs between SMN- and ACRN-WMs and between OFN- and FPN-WMs showed the high values under the movie condition. The dISFC ICC denoted high value between ON- and DN-WMs in the movie stimuli (Figure 7).

**Figure 8:**
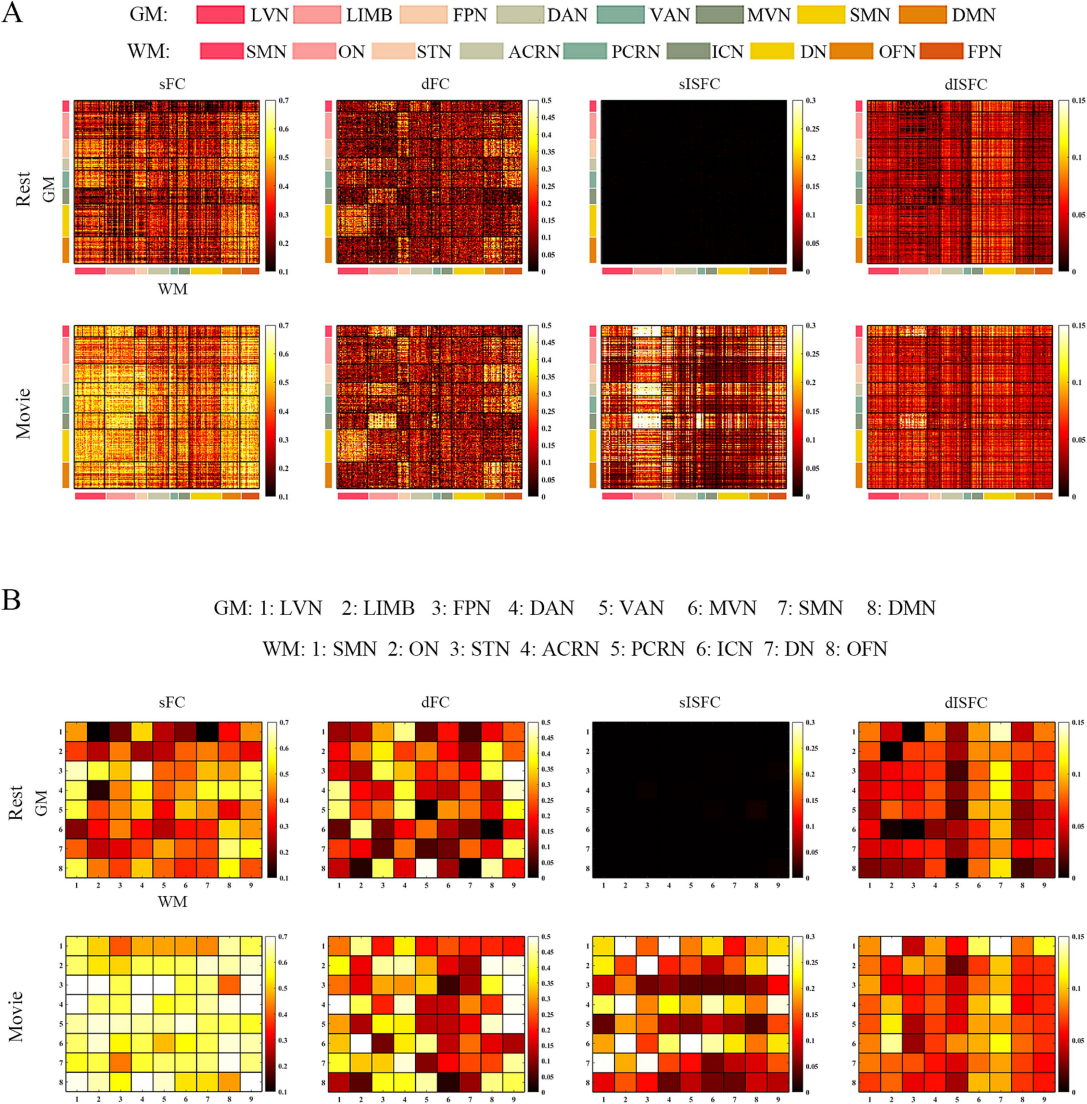
The ICC analysis of FC and ISFC between GM and WM. **(A)** ROI-level sFC, dFC, sISFC, and dISFC ICC matrices were calculated separately in the resting-state and movie-watching. **(B)** Network-level sFC, dFC, sISFC, and dISFC ICC matrices were calculated in the resting-state and the movie-watching separately. GM: LVN, Lateral visual network; LIMB, Limbic network; FPN, Frontoparietal network; DAN, Dorsal attention network; VAN, Ventral attention network; MVN, Medial visual network; SMN, Sensorimotor network; DMN, Default mode network; WM: SMN, Sensorimotor network; ON, Occipital network; STN, Superior temporal network; ACRN, Anterior corona radiata network; PCRN, Posterior corona radiata network; ICN, Inferior corticospinal network; DN, Deep network; OFN, Orbitofrontal network; FPN, Frontoparietal network.

For GM-WM, ROI-level sISFC ICC in DAN-ON was higher under the movie-watching than that during the resting-state. Generally, the sISFC reliability of GM-WM networks was enhanced by the naturalistic condition in network-level, especially in SMN-SMN, DAN-ON, LIMB-STN, LVN-ACRN, SMN-STN, MVN-PCRN, LIMB-FPN, and DAN-FPN. The dFC values of DMN-PCRN and FPN-FPN exhibited higher reliability than others in the resting-state. dISFC ICC of LVN-DN performed relatively high reliability under the movie-watching compared with the resting-state and other networks (Figure 8).

Permutation analysis was performed between the movie-watching ICC matrix and the resting-state ICC matrix to evaluate the reliability differences between two conditions. As the result shown, most of FC and ISFC indicated *p* < 0.0001. The biggest *p* value was in network-level GM dFC ICC matrices between the movie-watching and the resting-state, which was around with 0.01 (Supplementary Table S3).

### 3.7. Association between movie-watching and resting-state

**Figure 9:**
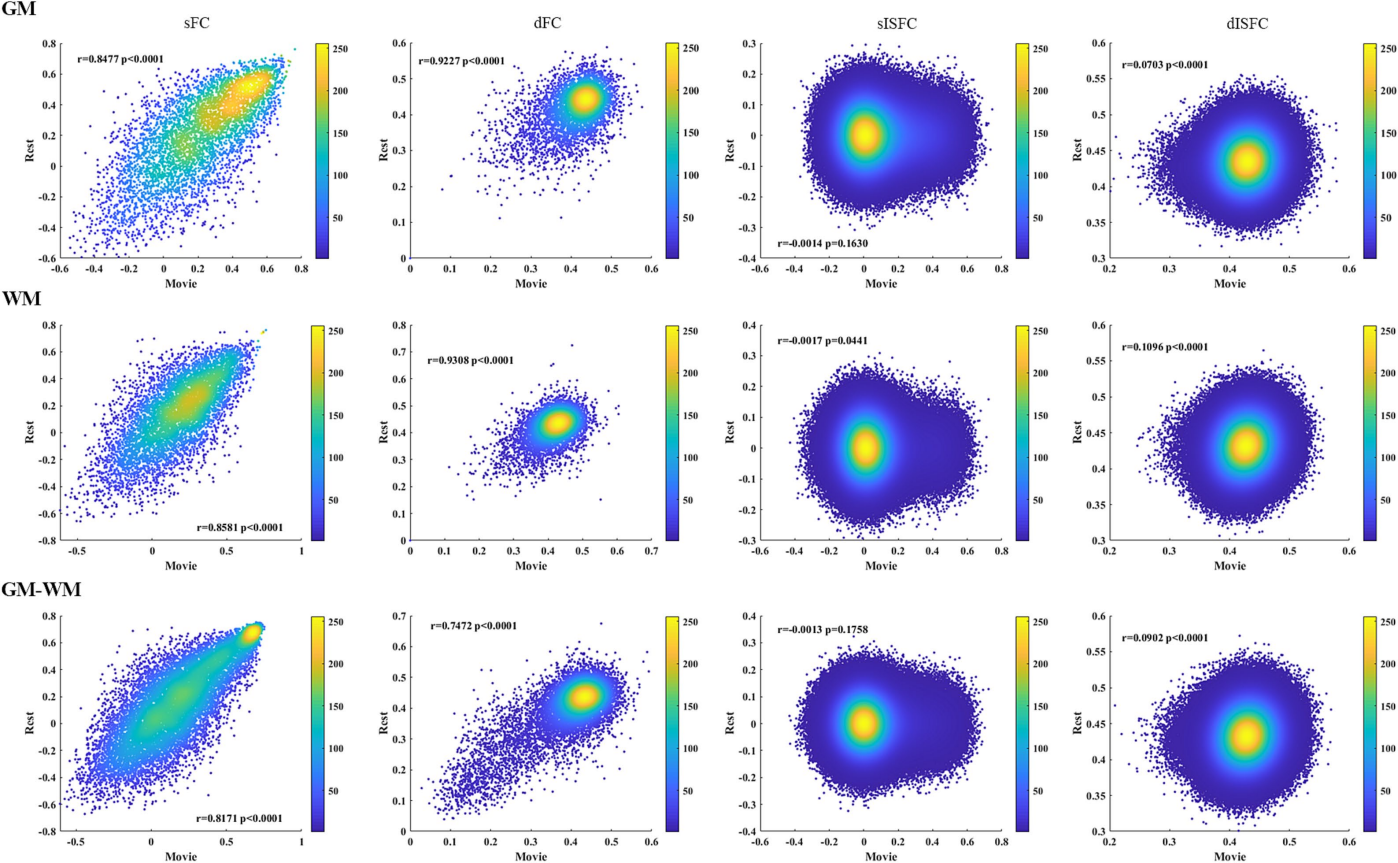
Heat maps of sFC, dFC, sISFC, and dISFC between the resting-state and the movie-watching in GM, WM, and GM-WM. We calculated the Pearson correlation coefficients for all connectivity indices between the resting-state and the naturalistic viewing. The densities were evaluated based on these correlation coefficients by using circles method.

We calculated the heat maps to estimate the associations between the resting-state and the movie-watching using both static and dynamic connectivity indices. Positive correlations for sFC were observed between the movie-watching and the resting-state in GM, WM, and GM-WM. Moreover, dFC also showed a degree of positive correlation between the movie condition and the resting-state. For sISFC and dISFC associations between naturalistic condition and resting-state, there were no correlations in GM, WM, and GM-WM (Figure 9).

**Figure 10:**
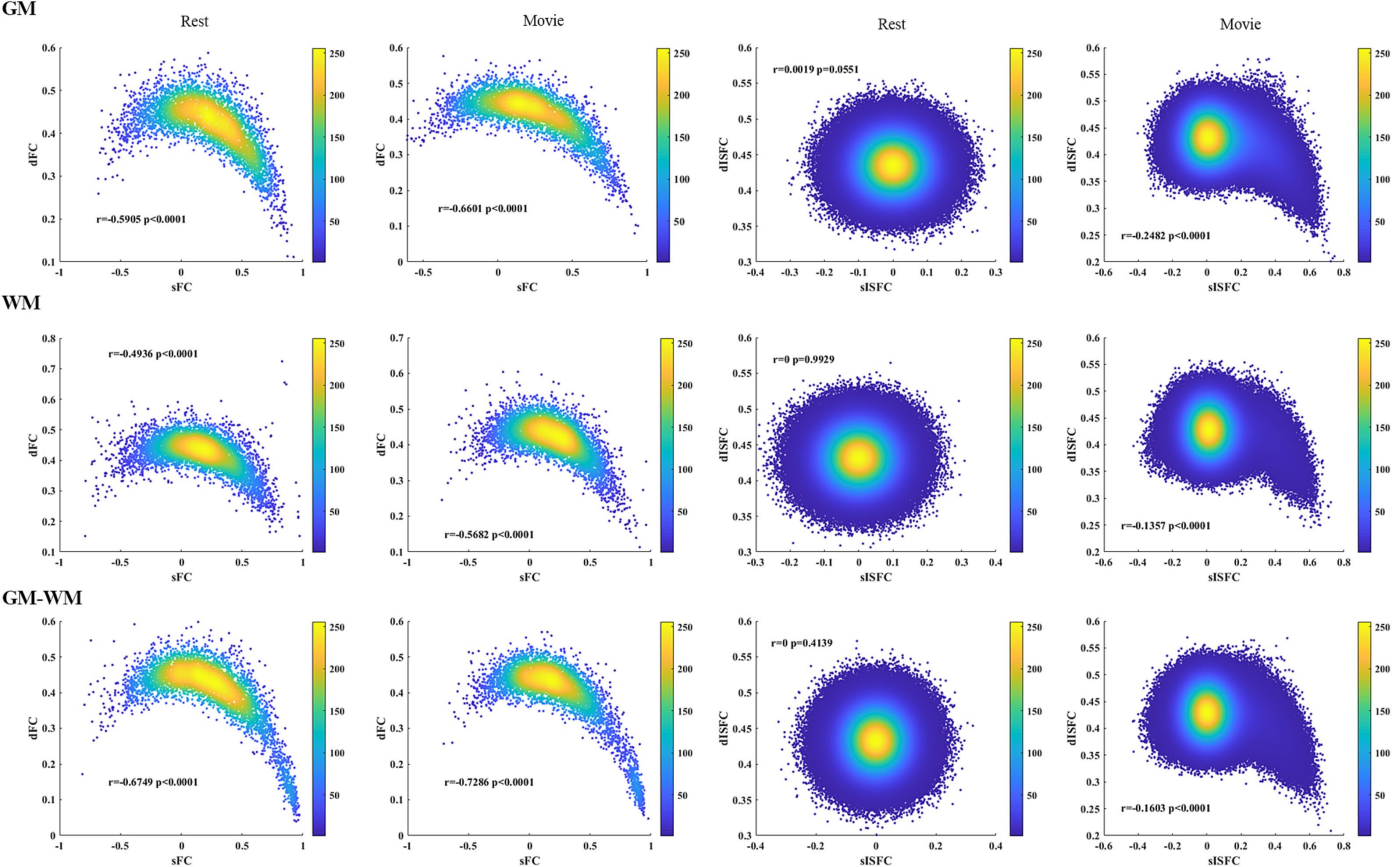
Heat maps of the resting-state and the movie-watching between sFC and dFC and between sISFC and dISFC in GM, WM, and GM-WM. Pearson correlation coefficients were performed between sFC and dFC as well as between sISFC and dISFC. The densities were evaluated based on these correlation coefficients by using circles method.

Associations between sFC and dFC in the resting-state and the movie-watching showed negative correlations in GM, WM, and GM-WM. In addition, we did not observe any correlations between sISFC and dISFC under the resting-state, but a relatively negative correlation was observed between sISFC and dISFC in the movie-watching (Figure 10).

## 4. Discussion

The current study compared the spatiotemporal characteristics of whole-brain FC between the naturalistic and the resting-state conditions. We found that the naturalistic stimuli not only improved sFC within and between networks compared with the resting-state, but lower sFC values also existed in some networks during the movie-watching. dFC values demonstrated opposite in general. The movie-watching did not change the FC patterns compared with the resting-state. sISFC was enhanced by the naturalistic condition, but the movie-watching had limited effect on dISFC, especially for ROI-level. The naturalistic paradigm improved the reliabilities of sFC, dFC, sISFC, and dISFC. Moreover, sFC and dFC showed positive correlations between the two conditions. sISFC and dISFC had negative correlation under the naturalistic viewing, but they demonstrated no correlation during the resting-state.

We found the high consistency patterns between the resting-state and movie-watching conditions similar to previous studies about the rfMRI and tfMRI (Cole et al., 2014; Krienen et al., 2014; Gratton et al., 2016). They suggested that the patterns of the functional networks were relatively stable, and the tasks or naturalistic condition only influenced the strength of FC. Lynch and colleagues denoted that all of the significant connectivity differences were stronger under the resting-state than that under the movie-watching, particularly for visual networks (Lynch et al., 2018). However, several weaker connections were observed in the current study under the resting-state than them during the naturalistic condition. Further, the increase and decrease in connectivity occurred in both primary and high-level GM networks, and superficial, middle, and deep WM layers. This denotes the influence of the naturalistic viewing for connectivity is not dependent on the traditional classifications of primary and high-level networks in GM and layers in WM, but the effect of the movie-watching for FC relates to the whole-brain. The explanations could be that: (1) The naturalistic condition contains a wealth of information, which induced different effect for different functional networks. (2) Functional networks might affect each other (Demirci et al., 2009). Tian and colleagues found considerable resemblance among movie-watching FC based on different movies (Tian et al., 2021), suggesting that the type of movie has limited impact on FC.

The higher ISFC under the naturalistic viewing was found to be associated with visual networks. Specifically, sISFC of LVN, MVN, ON-WM, and MVN-ON was improved during the movie-watching, demonstrating that ON-WM may show more similarly visual function to MVN than LVN. We found the sISFC within STN-WM was improved during the naturalistic viewing as STN-WM could be consider as the hub for the distributed brain network in complex stimuli (Jefferys et al., 2012; Lahnakoski et al., 2012). The movie-watching condition reduced dISFC values of MVN, ON-WM, and MVN-ON at the network-level. But for other networks at the network-level and all networks at the ROI-level, the naturalistic viewing performed limited effect about dISFC.

As the number of time points in MOVIE DAY1 and MOVIE DAY2 are different, we balanced the number of time points between them by extracting the first 1,596 time points of MOVIE DAY1, and compared the difference matrix of FC and ISFC. The results showed that there was limit change of FC and ISFC after the balance in the whole-brain (Supplementary Figures S3–S5).

The reliability of FC and ISFC was evaluated by ICC. In general, ICC values of the movie-watching were higher than them of the resting-state, denoting that the naturalistic viewing could improve the reliability of FC and ISFC. In addition, previous studies have demonstrated that ICCs of sFC were stronger than that of dFC within whole-brain under the resting-state (Wang et al., 2021). In our study, we also found that the reliability of sFC was higher than that of dFC under the naturalistic condition. Further reliability analysis showed the low reliability corresponding to sISFC and dISFC under the resting-state, denoting that ISFC may not be suitable for the resting-state due to the huge variance between subjects in the resting-state. The reason may be that subjects may have different thoughts under the rest scanning inducing the high signal fluctuation between subjects. Therefore, ISFC values were lower in the resting-state than those under the movie-watching. Though ISFC maps showed relative reliability in the naturalistic condition, ISFC ICC values were weaker than FC ICC values under the naturalistic viewing. The reason may be that FC is affected by more noise than ISFC, as ISFC could isolate the noise (Simony et al., 2016).

Chen and colleagues suggested nonparametric approaches and parametric methods for ISC (Chen et al., 2016, 2017; Chen G. et al., 2020). As these statistical approaches were designed for ISC and nonparametric approaches were easier to be performed, we determined SWB as the statistics of ISFC to detect the significantly movie-evoked connectivity. However, no significant ISFC was observed including visual networks, suggesting that though the naturalistic paradigm could enhance the inter-subject synchronization, the enhancement is not strong enough to pass the statistics. The reason may be that different subjects have different responses to the complex movie stimuli. Furthermore, non-significance of ISFC may also be because that the statistics approaches of ISC are not suitable for ISFC. ISFC is calculated between all of ROIs or networks across subjects, but ISC is evaluated between subjects in a same ROI or network.

The heat maps were performed to estimate the associations between static and dynamic properties during two conditions and the associations of connectivity indices between the naturalistic viewing and the resting-state. We found positive correlations between sFC of the resting-state and sFC of the naturalistic condition in GM, WM, and GM-WM. We also observed the relatively positive correlations between dFC of two conditions in whole-brain, suggesting a degree of similarity of FC between the resting-state and the movie-watching. Furthermore, both sISFC and dISFC showed no correlations between the two conditions, as there was scarcely any ISFC between subjects under the resting-state. The resting-state and the movie-watching showed similarly negative correlations between sFC and dFC in GM, WM, and GM-WM. We did not find any correlation between sISFC and dISFC under the resting-state, but sISFC values had weakly negative correlations with dISFC values under the naturalistic paradigm in whole-brain, suggesting that the movie-watching enhanced ISFC compared with the resting-state. However, considering the statistic results and the range of ISFC values, the enhancement is relatively weak.

Previous study has shown that even though movie contents were different in different scanning runs, there was considerable resemblance among movie-watching FC based on different movies (Tian et al., 2021). The similarity was also demonstrated in our study, and the most obvious networks were visual networks in gray matter and occipital network in white matter. However, the functional networks connectivity was reasonably considered to be influenced by the movie content. In HCP data, MOVIE1 and MOVIE3 contained clips from independent films (both fiction and documentary) made freely available under Creative Commons license on Vimeo. MOVIE2 and MOVIE4 contained clips from Hollywood films including action, adventure, science fiction, biography, drama, crime, thriller, and so on. In this study, we combined MOVIE1 and MOVIE2 as MOVIE Day1, and combined MOVIE3 and MOVIE4 as MOVIE Day2. The different movie types between MOVIE Day1 and MOVIE Day2 were science fiction (Day1), crime (Day1), thriller (Day1), comedy (Day2), family (Day2), and fantasy (Day2). The ICC values of LIMN, DAN, STN-WM, and OFN-WM were lower than other networks. LIMN could mediate emotional regulation and reward processing (Chen Y. L. et al., 2020). There was an evidence for a modulatory role of the DAN on the orienting of attention in space (Ptak and Schnider, 2010). The correlation between STN-WM and LIMN was higher than 0.8. Therefore, STN-WM probably has the similar function as LIMN. OFN-WM performed over 0.7 correlation coefficient with DMN that works in unison with language networks at certain points in the narrative, while exhibiting antagonistic responses at other times (Simony et al., 2016). Overall, as these networks demonstrated emotion, attention, and so on brain functions, different types of movie clips might induce different signals in a brain. Therefore, the low ICC values of these networks might be affected by different scanning runs and different movie types.

In this study, there are some limitations. (1) When subjects were watching movies, the brains were in a higher arousal condition with less head motion (Vanderwal et al., 2017). The effect of the noise should be evaluated about connectivity between the resting-state and the movie-watching in the following studies. (2) In this study, the subject studied were mostly between 22 and 35 years of age and did not cover the entire life span. It is quite possible, as has been shown in recent papers, that there are differences in sFC and dFC as the age range is increased and across gender (Jiang et al., 2020; Wen et al., 2020; Sen and Parhi, 2021; Snyder et al., 2021; Di and Biswal, 2022). Thus, it is quite possible that as a greater age range is used the systematic differences of the spatiotemporal characteristic of FC may be present. (3) Whether parametric method is better than nonparametric method for ISFC, and whether the influence between ROIs or networks of different subject should be consider in the parametric statistics, they are needed to be clarified in the future studies. (4) As the FC metrics were calculated based on data obtained from Caucasian populations, therefore there is potential influence when performing same calculations to Chinese populations (or other ethnic populations). Ge and colleges have studied the influence of populations about brain function between Chinese and people living in Western countries that from HCP dataset, and found that the corresponding large-scale brain parcellations were highly reproducible across the two datasets, with the language processing task showing the largest differences (Ge et al., 2022). However, whether ISFC will be influenced by populations, it need to be clarified in the further. Also, we did not focus on gender differences, thus FC may show variations after regressing them. (5) As some windows spanned two different runs, there is a potential effect about dFC and dISFC values.

## 5. Conclusion

This study investigated the effect of naturalistic viewing for whole-brain FC and ISFC, including sFC, dFC, sISFC, and dISFC compared with the resting-state. And we also evaluated the reliability of FC and ISFC in two conditions. Moreover, we explored the associations between static and dynamic properties and the associations between the naturalistic and the resting-state conditions. Specifically, the movie-watching not only improved FC values of inter- and intra-networks, but the decrease also exited. Besides, ISFC was enhanced generally under the naturalistic viewing. In this study, we did not find any ROI or network passed statistics of SWB for ISFC. The naturalistic paradigm generally enhanced reliabilities of sFC, dFC, sISFC, and dISFC compared with the resting-state, especially for sISFC and dISFC. Finally, the resting-state was positive correlation with the naturalistic viewing for sFC and dFC. Furthermore, sFC had negative correlation with dFC under the resting-state and the movie-watching. sISFC also showed relatively weak negative correlation with dISFC in the naturalistic viewing. As there was no significant ISFC and the range of ISFC values was small, the movie-watching has limit improvement for ISFC in this study.

## Data availability statement

The original contributions presented in the study are included in the article/Supplementary material, further inquiries can be directed to the corresponding authors.

## Ethics statement

The studies involving humans were approved by Washington University—University of Minnesota Consortium of the Human Connectome Project. The studies were conducted in accordance with the local legislation and institutional requirements. Written informed consent for participation in this study was provided by the participants’ legal guardians/next of kin. Written informed consent was obtained from the individual(s), and minor(s)' legal guardian/next of kin, for the publication of any potentially identifiable images or data included in this article.

## Author contributions

PH designed the study and wrote the original draft. PH, PW, RZ, and HY conducted the data analysis. PW and BB reviewed and edited the manuscript. All authors contributed to the article and approved the submitted version.

## Footnotes

^1^ http://humanconnectomeproject.org

^2^ http://rfmri.org/DPARSF

^3^ http://www.fil.ion.ucl.ac.uk/spm
