# Supplementary material for: Characterizing the spatiotemporal features of functional connectivity across the white matter and gray matter during the naturalistic condition
Source: Front Neurosci. 2023 Nov 9;17:1248610. doi: 10.3389/fnins.2023.1248610 (PMC10665512; doi:10.3389/fnins.2023.1248610)
Supplement: Supplementary file 1 [file Data_Sheet_1.docx]

**Characterizing the Spatiotemporal Features of Functional Connectivity across the White Matter and Gray Matter during the Naturalistic Condition**

**Peng Hu^1^, Pan Wang^1*^, Rong Zhao^1^, Hang Yang^2^, Bharat B Biswal^1, 3*^**

1, The Clinical Hospital of Chengdu Brain Science Institute, MOE Key Laboratory for Neuroinformation, Center for Information in Medicine, School of Life Science and Technology, University of Electronic Science and Technology of China, Chengdu, China

2, Chinese Institute for Brain Research, Beijing, China

3, Department of Biomedical Engineering, New Jersey Institute of Technology, Newark, NJ, 07102, USA

* Corresponding author:

Address correspondence to Pan Wang, PhD., The Clinical Hospital of Chengdu Brain Science Institute, MOE Key Laboratory for Neuroinformation, Center for Information in Medicine, School of Life Science and Technology, University of Electronic Science and Technology of China, Chengdu, China; E-mail: wpjoepan@163.com

Address correspondence to Bharat B. Biswal, PhD., 607 Fenster Hall, University Height, Newark, NJ, 07102, USA; E-mail: [bbiswal@gmail.com](mailto:bbiswal@gmail.com)

**
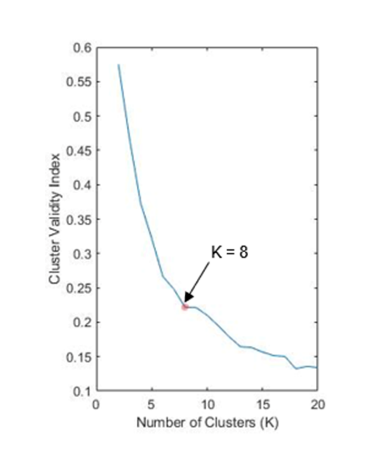
**

**Figure S1.** Determining the number of GM networks. Elbow criterion was used to decide the number of clusters (networks). The cluster validity index was the ratio between within-cluster to between-cluster distance. 8 clusters were determined as the results shown.

To compare between the results of clustering and traditional networks, we decided the Yeo’s 7 GM networks as the traditional GM networks and the Peer’s 12 WM networks as the traditional WM networks. The Dice Coefficient was determined as the similarity algorithm. In short, each of GM or WM clustering networks’ indices (space locations) were overlapped with each of GM or WM traditional networks’ indices in space. The higher the degree of spatial overlap, the stronger the similarity. (Figure S2).


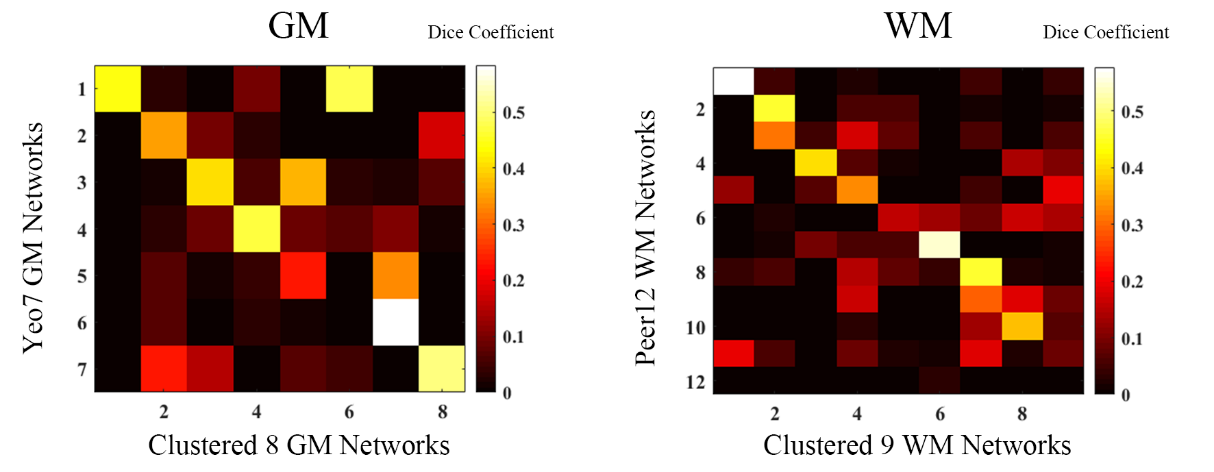


**Figure S2.** The similarity between traditional networks and clustered networks. The similarity was calculated by Dice Coefficient. The Yeo7 GM networks was set as the traditional GM networks. The Peer12 WM networks was decided as the traditional WM networks. Yeo7 GM networks: 1. Visual network; 2. Limbic network; 3. Frontoparietal network; 4. Dorsal attention network; 5. Ventral attention network; 6. Somatomotor network; 7. Default network. Clustered GM networks: 1. Lateral visual network; 2. Limbic network; 3. Frontoparietal network; 4. Dorsal attention network; 5. Ventral attention network; 6. Medial visual network; 7. Sensorimotor network; 8. Default mode network. Peer12 WM networks: 1. Sensorimotor superficial white-matter system; 2. Visual superficial white-matter system; 3. Inferior longitudinal fasciculus system; 4. Uncinate and middle temporal lobe tracts; 5. Ventral frontoparietal tracts; 6. Cingulum and associated tracts; 7. Inferior corticospinal tract; 8. Superior longitudinal fasciculus system; 9. Deep frontal white matter; 10. Forceps minor system; 11. Dorsal frontoparietal tracts; 12. Posterior cerebellar tracts . Clustered 9 WM networks: 1. Sensorimotor network; 2. Occipital network; 3. Superior temporal network; 4. Anterior corona radiata network; 5. Posterior corona radiata network; 6. Inferior corticospinal network; 7. Deep network; 8. Orbitofrontal network; 9. Frontoparietal network.


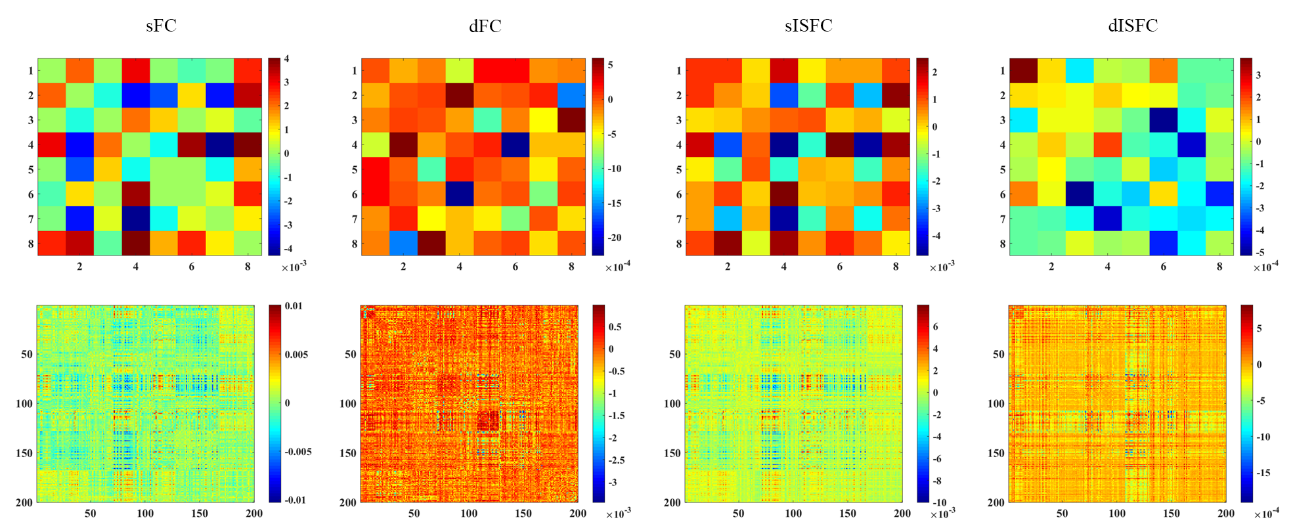


**Figure S3.** The different FC and ISFC matrices after balance time points between MOVIE DAY1 and MOVIE DAY2 within gray matter.


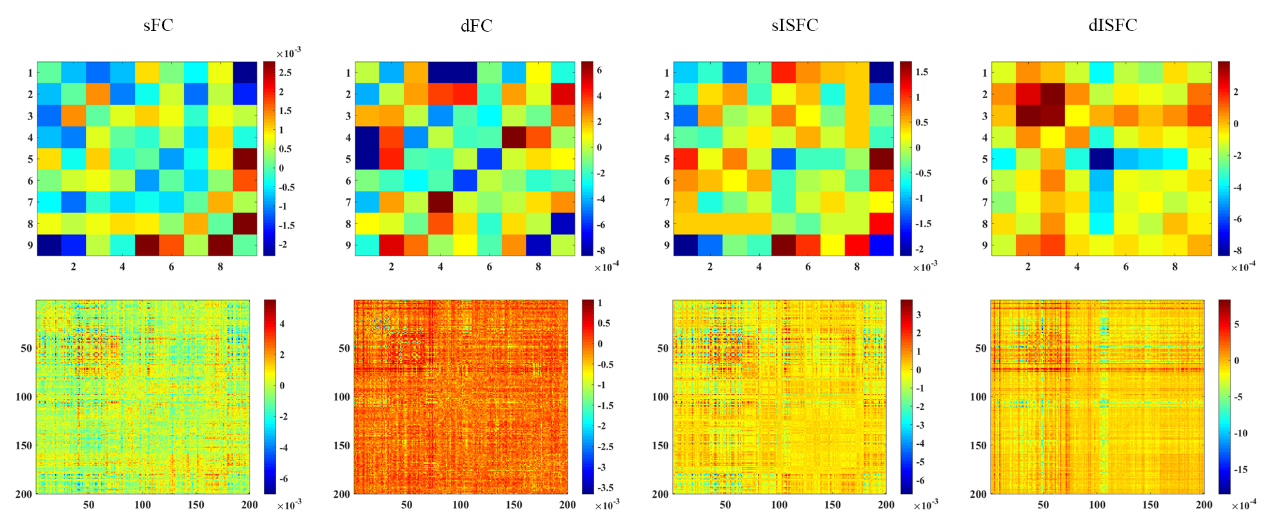


**Figure S4.** The different FC and ISFC matrices after balance time points between MOVIE DAY1 and MOVIE DAY2 within white matter.


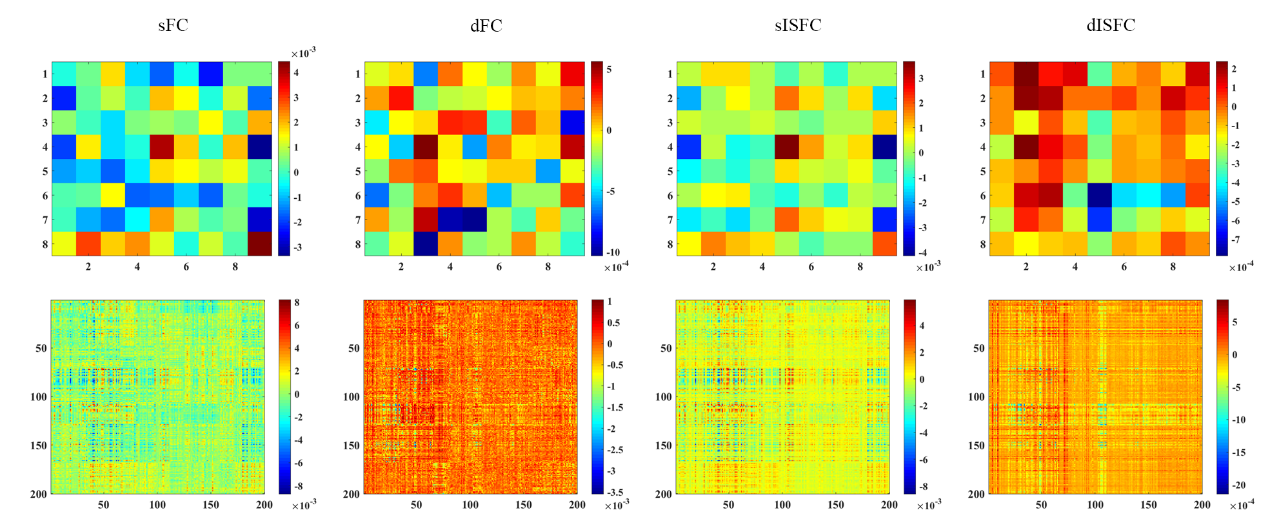


**Figure S5.** The different FC and ISFC matrices after balance time points between MOVIE DAY1 and MOVIE DAY2 between gray matter and white matter.

**Table S1.** sFC values between GM and WM networks in the resting-state. GM: 1. lateral visual network (LVN); 2. limbic network (LIMNLIMB); 3. frontoparietal network (FPN); 4. dorsal attention network (DAN); 5. ventral attention network (VAN); 6. medial visual network (MVN); 7. sensorimotor network (SMN); 8. default mode network (DMN). WM: 1. sensorimotor network in WM (SMN-WM); 2. occipital network in WM (ON-WM); 3. superior temporal network in WM (STN-WM); 4. anterior corona radiata network in WM (ACRN-WM); 5. posterior corona radiata network in WM (PCRN-WM); 6. inferior corticospinal network in WM (ICN-WM); 7. deep network in WM (DN-WM); 8. orbitofrontal network in WM (OFN-WM); 9. frontoparietal network in WM (FPN-WM).

| WM  GM | 1 | 2 | 3 | 4 | 5 | 6 | 7 | 8 | 9 |
| --- | --- | --- | --- | --- | --- | --- | --- | --- | --- |
| 1 | 0.01 | 0.63 | 0.04 | **0.44** | 0.04 | **0.15** | **0.24** | 0.08 | 0.14 |
| 2 | 0.36 | 0.24 | **0.82** | 0.19 | 0.30 | -0.16 | -0.10 | 0.40 | 0.44 |
| 3 | -0.08 | 0.03 | 0.25 | 0.10 | 0.06 | -0.17 | -0.02 | 0.68 | 0.60 |
| 4 | 0.37 | 0.59 | 0.07 | 0.22 | 0.10 | -0.21 | 0.03 | 0.02 | 0.51 |
| 5 | 0.36 | 0.22 | 0.12 | 0.28 | -0.14 | -0.28 | -0.12 | 0.21 | **0.84** |
| 6 | 0.40 | **0.84** | 0.14 | 0.30 | 0.36 | -0.10 | 0.10 | -0.02 | 0.19 |
| 7 | **0.91** | 0.34 | 0.19 | 0.30 | -0.01 | -0.28 | -0.12 | -0.18 | 0.40 |
| 8 | -0.13 | -0.04 | 0.57 | -0.02 | **0.48** | -0.06 | 0.00 | **0.77** | 0.11 |

**Table S2.** sFC values between GM and WM networks in the movie-watching. GM: 1. lateral visual network (LVN); 2. limbic network (LIMNLIMB); 3. frontoparietal network (FPN); 4. dorsal attention network (DAN); 5. ventral attention network (VAN); 6. medial visual network (MVN); 7. sensorimotor network (SMN); 8. default mode network (DMN). WM: 1. sensorimotor network in WM (SMN-WM); 2. occipital network in WM (ON-WM); 3. superior temporal network in WM (STN-WM); 4. anterior corona radiata network in WM (ACRN-WM); 5. posterior corona radiata network in WM (PCRN-WM); 6. inferior corticospinal network in WM (ICN-WM); 7. deep network in WM (DN-WM); 8. orbitofrontal network in WM (OFN-WM); 9. frontoparietal network in WM (FPN-WM).

| WM GM | 1 | 2 | 3 | 4 | 5 | 6 | 7 | 8 | 9 |
| --- | --- | --- | --- | --- | --- | --- | --- | --- | --- |
| 1 | 0.05 | 0.78 | 0.16 | **0.46** | 0.13 | **0.09** | **0.13** | -0.04 | 0.16 |
| 2 | 0.30 | 0.30 | **0.84** | 0.37 | 0.15 | 0.00 | -0.09 | 0.24 | 0.53 |
| 3 | -0.11 | -0.08 | 0.06 | 0.04 | 0.18 | -0.13 | 0.07 | 0.72 | 0.53 |
| 4 | 0.30 | 0.66 | 0.07 | 0.30 | 0.12 | -0.14 | 0.03 | -0.03 | 0.49 |
| 5 | 0.28 | 0.08 | 0.19 | 0.26 | 0.03 | -0.18 | -0.03 | 0.38 | **0.79** |
| 6 | 0.23 | **0.87** | 0.09 | 0.36 | **0.40** | -0.03 | 0.11 | -0.02 | 0.23 |
| 7 | **0.88** | 0.14 | 0.25 | 0.35 | 0.00 | -0.11 | -0.03 | -0.12 | 0.34 |
| 8 | -0.10 | 0.00 | 0.49 | 0.10 | **0.40** | -0.04 | 0.04 | **0.77** | 0.32 |

**Table S3.** Permutation p values of ICC between the movie-watching and the resting-state. Permutation method was performed between the movie-watching ICC matrix and the resting-state ICC matrix. The number of iterations was 5000. The counts were added when the raw ICC difference between two conditions was higher than the shuffle ICC difference between two conditions. The p values were calculated by the counts divided by the iteration number.

|  | sFC | | dFC | | sISFC | | dISFC | |
| --- | --- | --- | --- | --- | --- | --- | --- | --- |
|  | Network | ROI | Network | ROI | Network | ROI | Network | ROI |
| GM | p<0.0001 | p<0.0001 | p=0.0176 | p<0.0001 | p<0.0001 | p<0.0001 | p<0.0001 | p<0.0001 |
| WM | p<0.0001 | p<0.0001 | p=0.0032 | p<0.0001 | p<0.0001 | p<0.0001 | p=0.0022 | p<0.0001 |
| GM-WM | p<0.0001 | p<0.0001 | p=0.0028 | p<0.0001 | p<0.0001 | p<0.0001 | p<0.0001 | p<0.0001 |
